# Targeting LOXL4-driven matrix stiffening with acetyldigoxin restores CD8^+^ T cell function in lung cancer

**DOI:** 10.1126/sciadv.aef1694

**Published:** 2026-09-11

**Authors:** Xuyu Gu, Yifei Zhu, Li Xu, Xinnan Xu, Kaiqi Jin, William C. Cho, Qiyu Fang

**Affiliations:** ^1^ Department of Oncology, Shanghai Pulmonary Hospital, School of medicine, Tongji University, Shanghai 200433, China.; ^2^ Department of Pancreatic Surgery, Fudan University Shanghai Cancer Center, Shanghai 200032, China.; ^3^ Department of Oncology, Shanghai Medical College, Fudan University, Shanghai 200032, China.; ^4^ Department of Thoracic Surgery, Shanghai Pulmonary Hospital, School of medicine, Tongji University, Shanghai 200433, China.; ^5^ Department of Clinical Oncology, Queen Elizabeth Hospital, Hong Kong SAR, China.

## Abstract

Extracellular matrix (ECM) stiffness is known to impair T cell function, yet the underpinning molecular cascade remains undefined. This paper investigates the role of lysyl oxidase–like 4 (LOXL4) in ECM stiffening and CD8^+^ T cell function in lung cancer. *Loxl4* knockout and mouse recombinant LOXL4 protein systems, along with *Piezo1*, *Ybx1*, *Acly*, and *Kat2a* conditional knockout mouse models were established. ECM stiffness was measured by atomic force microscopy, and T cell exhaustion markers were analyzed using flow cytometry. RNA sequencing, ATAC-seq, CUT&Tag, chromatin immunoprecipitation–quantitative polymerase chain reaction, and luciferase assays were used to explore the underlying molecular mechanisms. Molecular docking was performed to explore Food and Drug Administration–approved agents targeting LOXL4. The results demonstrated that tumor-derived LOXL4 stiffened the ECM, which activates the mechanosensor Piezo1 in CD8^+^ T cells, triggering Ca^2+^ influx and downstream FAK1-YAP1 signaling. Nuclear YAP1 transactivated YBX1, which recruited the metabolic enzyme ACLY and the histone acetyltransferase KAT2A to exhaustion gene loci, epigenetically reinforcing terminal exhaustion. Conditional knockout of *Piezo1*, *Ybx1*, *Acly*, and *Kat2a* in murine CD8^+^ T cells abolished stiffness-induced exhaustion and suppressed tumor growth. Acetyldigoxin was identified as a high-affinity LOXL4 inhibitor. It softened the ECM, disrupted the mechanosignaling-epigenetic axis, reversed CD8^+^ T cell exhaustion, and synergized with anti–PD-1 blockade to achieve durable tumor regression. In conclusion, this study uncovers a mechanotransduction-to-epigenetic pathway where LOXL4-driven matrix stiffening induces CD8^+^ T cell exhaustion. Repurposing acetyldigoxin as a LOXL4-targeted therapy offers a promising clinical strategy to overcome ECM-mediated immunotherapy resistance in lung cancer.

## INTRODUCTION

Lung cancer ranked as the leading type in terms of new cases (12.4%) and the primary cause of cancer-related mortality (18.7%) across the globe according to 2022 statistics (*1*). Non–small cell lung cancer (NSCLC) accounts for roughly 85% of all lung cancer cases, among which lung adenocarcinoma (LUAD) (around 40%) and squamous cell carcinoma (about 30%) represent the predominant histological forms (*2*, *3*). Clinically, merely a minority of cases are identified at operable early stages permitting curative resection. In contrast, the overwhelming majority present with metastatic or locally advanced disease, necessitating systemic approaches including cytotoxic chemotherapy, radiotherapy, targeted agents, or immune-based therapies (*4*). Immune checkpoint blockade (ICB) can elicit deep and long-lasting tumor regressions and have been swiftly integrated into standard care for advanced NSCLC, thereby prolonging life expectancy in this setting (*5*, *6*). Regrettably, even with these therapeutic advances, long-term prognosis remains dismal, evidenced by a 5-year overall survival of just 26% (*7*). Such sobering figures underscore the pressing demand for innovative treatment modalities capable of circumventing resistance mechanisms and yielding superior clinical benefits against this lethal malignancy.

Oncology has progressed beyond a narrow focus on aberrantly proliferating neoplastic clones to recognizing tumors as intricate ecosystems integrating cancerous elements with a heterogeneous array of nonneoplastic cells and acellular constituents collectively known as the tumor stroma or tumor microenvironment (TME) (*8*). The extracellular matrix (ECM) serves as a foundational architecture that preserves tissue integrity under normal conditions while emerging as a pivotal TME element that facilitates oncogenesis (*9*, *10*). As tumors evolve, an elaborate ECM lattice forms through the assembly of fibrillar and nonfibrillar collagens, elastin fibers, proteoglycans, glycoproteins, fibronectins, laminins, and additional structural proteins (*11*, *12*). Heightened collagen accrual, the principal ECM constituent, is consistently documented in various solid malignancies and correlates with accelerated disease advancement (*13*). Beyond physically anchoring both malignant and stromal populations, the ECM functions as a depot for bioactive molecules. Moreover, it engages adjacent cells via receptor-mediated interactions, triggering signaling pathways that fuel proliferation, invasion, and distant colonization (*14*). Physiologically, ECM exhibits pliability and elasticity (*15*). Within desmoplastic tumors, however, persistent activation of cancer-associated fibroblasts coupled with lysyl oxidase (LOX)–driven covalent cross-linking provokes excessive matrix deposition and rigidity (*16*). This biomechanical alteration defines desmoplasia and markedly alters immune dynamics, typically fostering an immunotolerant milieu that impairs effector antitumor responses (*17*, *18*). Effector lymphocytes tasked with tumor eradication become functionally compromised amid this restructured matrix, which cultivates immunosuppression and erects a formidable barrier safeguarding neoplastic viability and dissemination (*19*).

Across diverse solid neoplasms, ECM reconfiguration typically involves augmented collagen biosynthesis and fibrillogenesis, paralleling by up-regulated expression of degradative and cross-linking enzymes including matrix metalloproteinases (MMPs), LOX, LOX-like isoforms (LOXLs), and related factors (*20*). Differential MMP levels within the TME often mirror tumor aggressiveness, underscoring their role in architectural reconfiguration during epithelial carcinogenesis, inclusive of pulmonary origins (*21*, *22*). LOX, an extracellular copper-reliant enzyme, mediates irreversible intermolecular bridges between collagen and elastin strands (*23*). The *LOX* gene family encompasses five paralogs: prototypical *LOX* and four *LOXL* variants (*LOXL1 to LOXL4*). Among these, LOXL4, the latest identified member, functions as an exported amine oxidase that enhances matrix rigidity and has lately been implicated in dampening T cell efficacy within solid cancers (*24*). The oncogenic versus suppressive roles of LOXL4 appear highly tissue- and context-specific, attracting growing research interest (*25*). Building on our previous finding that LNC511-mediated LOXL4 up-regulation facilitates LUAD progression, this study seeks to further elucidate LOXL4’s involvement in lung cancer evolution, with particular emphasis on modulation of ECM stiffness and CD8^+^ T cell performance. In addition, the investigation probes underlying mechanistic pathways and evaluates candidate compounds for therapeutic LOXL4 inhibition.

## RESULTS

### *Loxl4* or *Piezo1* knockout reduces tumorigenic activity of 3LL cells and enhances CD8^+^ T cell function in syngeneic tumor models

**Fig. 1.**
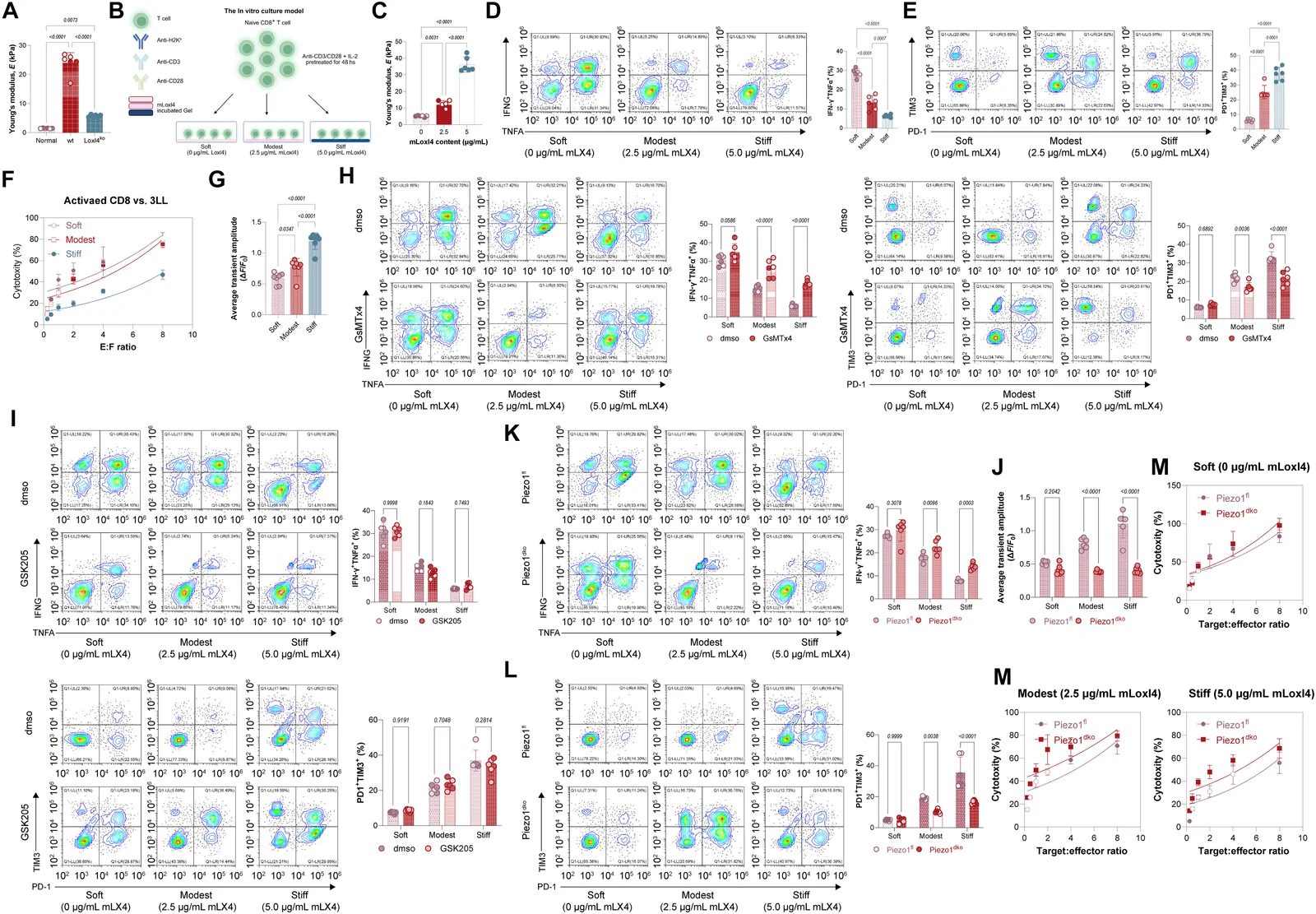
LOXL4 knockout reduces ECM stiffness and CD8^+^ T cell exhaustion, effects reproduced by Piezo1 deletion in CD8^+^ T cells. WT or *Loxl4*^ko^ mouse 3LL lung cancer cells were implanted into C57 mice subcutaneously to generate syngeneic tumors. (**A**) Average tissue stiffness in 3LL-formed tumors evaluated by elastic modulus measurement. (**B**) Schematic diagram of the in vitro culture model: mLX4 was therefore added to type I collagen at 0, 2.5, or 5 μg/ml to form gels, onto which αCD3/αCD28 antibody–stimulated CD8^+^ T cells were seeded. Created in BioRender. Gu, X. (2026) https://BioRender.com/3ayex8q. h, hours. (**C**) Average gel stiffness in mLX4-induced gels evaluated by elastic modulus measurement. (**D** and **E**) Populations of effector CD8^+^ T cells (TNFα^+^IFN-γ^+^) and exhausted T cells (PD-1^+^TIM-3^+^) after culture on gels across groups determined using flow cytometry. (**F**) Cytotoxicity assay of T cells against 3LL cells in vitro. (**G**) Quantification of average Ca^2+^ transient amplitude (Δ*F*/*F*_0_) in CD8^+^ T cells. (**H** and **I**) Addition of TRPV inhibitor GSK205 or Piezo inhibitor GsMTx4 to the culture system of activated T cells in gels, followed by flow cytometry analysis of effector CD8^+^ T cells (TNFα^+^IFN-γ^+^) and exhausted T cells (PD-1^+^TIM-3^+^) determined using follow cytometry; CD8^+^ T cells were extracted from *Piezo1*^dko^ or *Piezo1*^fl/fl^ mice and seeded on gels. (**J**) Quantification of average Ca^2+^ transient amplitude (Δ*F*/*F*_0_) in *Piezo1*^dko^ or *Piezo1*^fl/fl^ CD8^+^ T cells. (**K** and **L**) Populations of effector CD8^+^ T cells (TNFα^+^IFN-γ^+^) and exhausted T cells (PD-1^+^TIM-3^+^) of *Piezo1*^dko^ or *Piezo1*^fl/fl^ CD8^+^ T cells after culture on gels determined using follow cytometry. (**M**) Cytotoxicity assay of *Piezo1*^dko^ or *Piezo1*^fl/fl^ CD8^+^ T cells against 3LL cells after culture on gels. Data are presented as dot plots and bars. Statistical significance was determined using unpaired *t* tests (A), one-way analysis of variance (ANOVA), or two-way ANOVA followed by Sidak’s post hoc test [(C), (D), (E), (G), (F), and (H) to (M)]. Statistical significance was set at *P* < 0.05.

To examine the function of LOXL4 in lung cancer, wild-type (WT) or *Loxl4* knockout (*Loxl4*^ko^) mouse 3LL lung cancer cells were implanted into either immunocompromised BALB/c nude mice or immunocompetent C57 mice subcutaneously. It was observed that the growth of *Loxl4*^ko^ 3LL cells was significantly retarded in both C57 and nude mice (fig. S1, A to D). However, growth suppression was more pronounced in C57 mice (fig. S1E). Given that C57 mice have intact immunity, an alteration in the TME may be implicated in tumor-suppressive events upon *Loxl4* knockout. As predicted, increased effector CD8^+^ T cells [tumor necrosis factor–α (TNFα^+^) interferon-γ (IFN-γ^+^] and reduced exhausted T cells (PD-1^+^TIM-3^+^) were observed in tumor tissues in *Loxl4*^ko^ C57 mice (fig. S1, F and G). We then hypothesized that LOXL4, as a secretory amine oxidase acting on ECM substrates, influences CD8^+^ T cell function in tumor tissues by modulating ECM. A substantial reduction in Young’s modulus was detected in tumor tissues formed by *Loxl4*^ko^ 3LL cells in C57 mice (Fig. 1A), indicating a decrease in ECM stiffness. Notably, the stiffness of WT tumors was significantly higher than that of healthy nontumor lung tissue, which exhibited a baseline Young’s modulus of approximately 1.5 kPa. To study further, high-purity recombinant mouse LOXL4 protein (mLX4) was therefore added to type I collagen at 0, 2.5, or 5 μg/ml to form gels, onto which αCD3/αCD28 antibodies-stimulated CD8^+^ T cells were seeded (Fig. 1B). Higher mLX4 concentrations produced gels with greater stiffness (Fig. 1C). Increased gel stiffness correlated with decreased effector T cells (Fig. 1D), elevated numbers of exhausted CD8^+^ T cells (Fig. 1E), and significantly impaired CD8^+^ T cell cytotoxicity (Fig. 1F). Existing literature indicated that CD8^+^ T cells in the tumor or tissue microenvironment are affected by not only chemical signals (antigens and cytokines) but also to mechanical cues (matrix stiffness, shear force, compressive stress, and cell-ECM interaction forces) (*26*, *27*). On collagen-formed gel with 5 μg/ml mLX4, Ca^2+^ influx in T cells was markedly increased (Fig. 1G). Furthermore, the TRPV4 (transient receptor potential vanilloid 4, a mechanosensitive Ca^2+^-permeable channel) inhibitor GSK205 and Piezo (a stretch-activated Ca^2+^ channel exquisitely sensitive to matrix rigidity) inhibitor GsMTx4 were added to the coculture system. GsMTx4 markedly suppressed ECM stiffness–induced CD8^+^ T cell exhaustion. Whereas the TRPV inhibitor led to a visible minor suppression, it did not show a significant effect (*P* > 0.05) (Fig. 1, H and I). This suggests that while TRPV4 may contribute marginally to the mechanical sensing of the ECM, it is not the primary driver of stiffness-induced exhaustion in this model.

Thus, LOXL4-mediated ECM stiffness was hypothesized to activate Piezo in response to mechanical stress, promoting Ca^2+^ influx and subsequent CD8^+^ T cell exhaustion. To verify this hypothesis, *Piezo1*^fl/fl^ and *Cd8a*-Cre mice were acquired and allowed to mate to generate a CD8^+^ T cell–specific *Piezo1* knockout (*Piezo1*^dko^) strain. In the setting of subcutaneous injection, the growth of 3LL cells was substantially restricted in *Piezo1*^dko^ compared to *Piezo1*^fl/fl^ mice (fig. S2, A and B), accompanied by a decreased population of exhausted T cells (fig. S2C) and increased number of effector T cells (fig. S2D). Furthermore, CD8^+^ T cells were extracted from *Piezo1*^dko^ or *Piezo1*^fl/fl^ mice and seeded on gels as in Fig. 1B. T cells from *Piezo1*^dko^ mice exhibited minimal response to ECM stiffness (Fig. 1J) and markedly attenuated mLX4-mediated stiffness-induced CD8^+^ T cell exhaustion (Fig. 1, K to M). To determine whether tumor cells secret LOXL4 into the TME to activate Piezo1 in CD8^+^ T cells, promote Ca^2+^ influx, and induce exhaustion, mLX4 was injected intratumorally into *Piezo1*^dko^ or *Piezo1*^fl/fl^ mice receiving 3LL injection. This procedure significantly accelerated 3LL growth in *Piezo1*^fl/fl^ mice but had no substantial effect in *Piezo1*^dko^ mice (fig. S3, A and B). Moreover, mLX4 failed to significantly alter the populations of effector or exhausted CD8^+^ T cells in *Piezo1*^dko^ mice but markedly promoted CD8^+^ T cell exhaustion in *Piezo1*^dko^ mice (fig. S3, C and D). Furthermore, using a tissue microarray (TMA) comprising 104 LUAD cases, we observed that LOXL4 staining intensity was significantly higher in tumor tissues than in adjacent nontumor tissues (fig. S4A). In addition, the abundance of CD8^+^ T cells and GZMB^+^ cells in the tumor tissues of patients with high LOXL4 expression was significantly lower than that in the low-expression group (fig. S4, B and C). Consistently, analysis of the TCGA dataset revealed that LOXL4 expression is negatively correlated with the infiltration of CD8^+^ T cells and CTLs (fig. S4, D and E).

### LOXL4 regulates ECM stiffness to promote CD8^+^ T cell exhaustion through Piezo1-mediated FAK1-YAP1 axis

Subsequently, quantitative polymerase chain reaction (qPCR) analysis revealed markedly increased Piezo1 and Piezo2 levels in CD8^+^ T cells cultured on stiff gels, accompanied by increased nuclear YAP1 localization and FAK^Tyr397^ phosphorylation (fig. S5, A to C). Given that Piezo1 is the predominant mechanosensitive ion channel in immune cells and it showed more pronounced up-regulation in CD8^+^ T cells on stiff substrates, we selected Piezo1 for further investigation. Supplementation of Yoda1, a potent Piezo1 agonist, in CD8^+^ T cells seeded on soft or moderate gels (0 or 2.5 μg/ml mLX4) markedly enhanced Ca^2+^ influx (fig. S5D), suppressed effector T cell polarization (fig. S5E), exacerbated exhaustion (fig. S5F), and increased FAK^Tyr397^ phosphorylation and YAP1 nuclear entry (fig. S5G). However, in *Piezo1*^dko^ CD8^+^ T cells, ECM stiffness alterations did not affect YAP1-FAK1 activity (fig. S5, I and J). Thus, the FAK-YAP1 pathway was hypothesized to be responsible for CD8^+^ T cell exhaustion following LOXL4-mediated ECM stiffness.

**Fig. 2.**
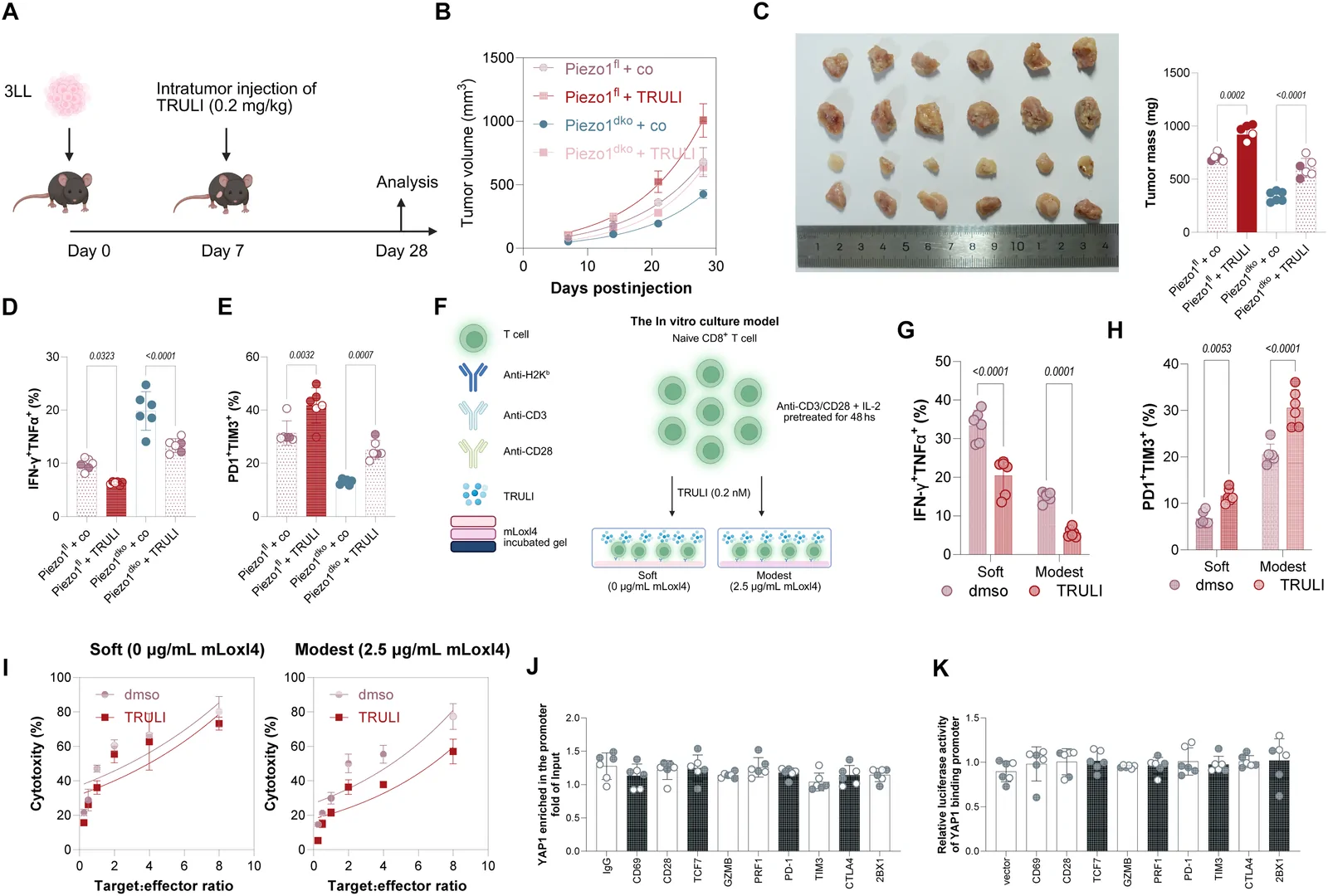
LOXL4 regulates ECM stiffness to promote CD8^+^ T cell exhaustion through Piezo1-mediated FAK1-YAP1 axis. (**A**) Schematic diagram of the intratumoral injection of TRULI (LATS1/2 inhibitor) into 3LL-tumors in *Piezo1*^dko^ or *Piezo1*^fl/fl^ mice on day 7. Created in BioRender. Gu, X. (2026) https://BioRender.com/xdbdblf. (**B**) Weekly volume change in subcutaneous tumors in mice receiving TRULI administration. (**C**) Representative images and weight of subcutaneous tumors in mice receiving TRULI administration on day 28. (**D** to **E**) Populations of effector CD8^+^ T cells (TNFα^+^IFN-γ^+^) and exhausted T cells (PD-1^+^TIM-3^+^) in tumors in *Piezo1*^dko^ or *Piezo1*^fl/fl^ mice receiving TRULI administration determined using flow cytometry. (**F**) Schematic diagram of the supplementation of TRULI in CD8^+^ T cells seeded on soft or moderate gels (0 or 2.5 μg/ml mM mLX4). Created in BioRender. Gu, X. (2026) https://BioRender.com/19x2q37. (**G** and **H**) Populations of effector CD8^+^ T cells (TNFα^+^IFN-γ^+^) and exhausted T cells (PD-1^+^TIM-3^+^) on gels after the TRULI supplementation determined using flow cytometry. (**I**) Cytotoxicity assay of T cells after the TRULI supplementation against 3LL cells in vitro. (**J**) ChIP with anti-YAP1 followed by qPCR to examine the binding of YAP1 to CD69, CD28, TCF7, GZMB, PRF1, PD-1, TIM-3, CTLA4, and TOX promoter segments. (**K**) Luciferase activity in 293T cells transfected with CD69, CD28, TCF7, GZMB, PRF1, PD-1, TIM-3, CTLA4, or TOX promoter-driven luciferase reporters and YAP1-expressing plasmids. For animal experiments, each group contained six mice. Cell experiments repeated six times. Data are presented as dot plots and bars. Statistical significance was determined one-way ANOVA followed by Tukey’s post hoc test [(C), (D), (E), (J), and (K)], or two-way ANOVA followed by Sidak’s post hoc test [(B), (G), (H) and (I)]. Statistical significance was set at *P* < 0.05.

To verify this hypothesis, TRULI (LATS1/2 inhibitor) was then injected into 3LL tumors in *Piezo1*^dko^ or *Piezo1*^fl/fl^ mice (Fig. 2A). The intratumoral administration TRULI significantly promoted tumor growth (Fig. 2, B and C), reduced effector T cells (Fig. 2D), and increased exhausted T cells (Fig. 2E). In parallel, TRULI supplementation in CD8^+^ T cells seeded on soft or moderate gels (0 or 2.5 μg/ml mLX4) (Fig. 2F) enhanced mLX4-induced CD8^+^ T cell exhaustion (Fig. 2, G to I).

To verify whether YAP1 activation led to CD8^+^ T cell exhaustion, levels of T cell function markers in CD8^+^ T cells cultured in soft, moderate, and stiff gels were determined, demonstrating substantial reductions in the expression of effector markers CD69, CD28, TCF7, GZMB, and PRF1 while increases in exhaustion indicators PD-1, TIM-3, CTLA4, and TOX (fig. S6A). In CD8^+^ T cells extracted from *Piezo1*^dko^, the levels of effector markers were up-regulated while the exhaustion markers were suppressed (fig. S6B), effects counteracted by the additional administration of TRULI in tumors (fig. S6C). Chromatin immunoprecipitation (ChIP)–qPCR with anti-YAP1 revealed minimal binding to the promoter of these genes (Fig. 2J), and luciferase assay results denied a direct influence of YAP1 on the transcription activity of these genes (Fig. 2K). These results suggested that FAK1-YAP1 possibly interacts with other transcription modulators to mediate CD8^+^ T cell exhaustion in response to LOXL4-induced ECM stiffening.

### YBX1 is activated by YAP1 and participates in mechanical force–responsive CD8^+^ T cell exhaustion

**Fig. 3.**
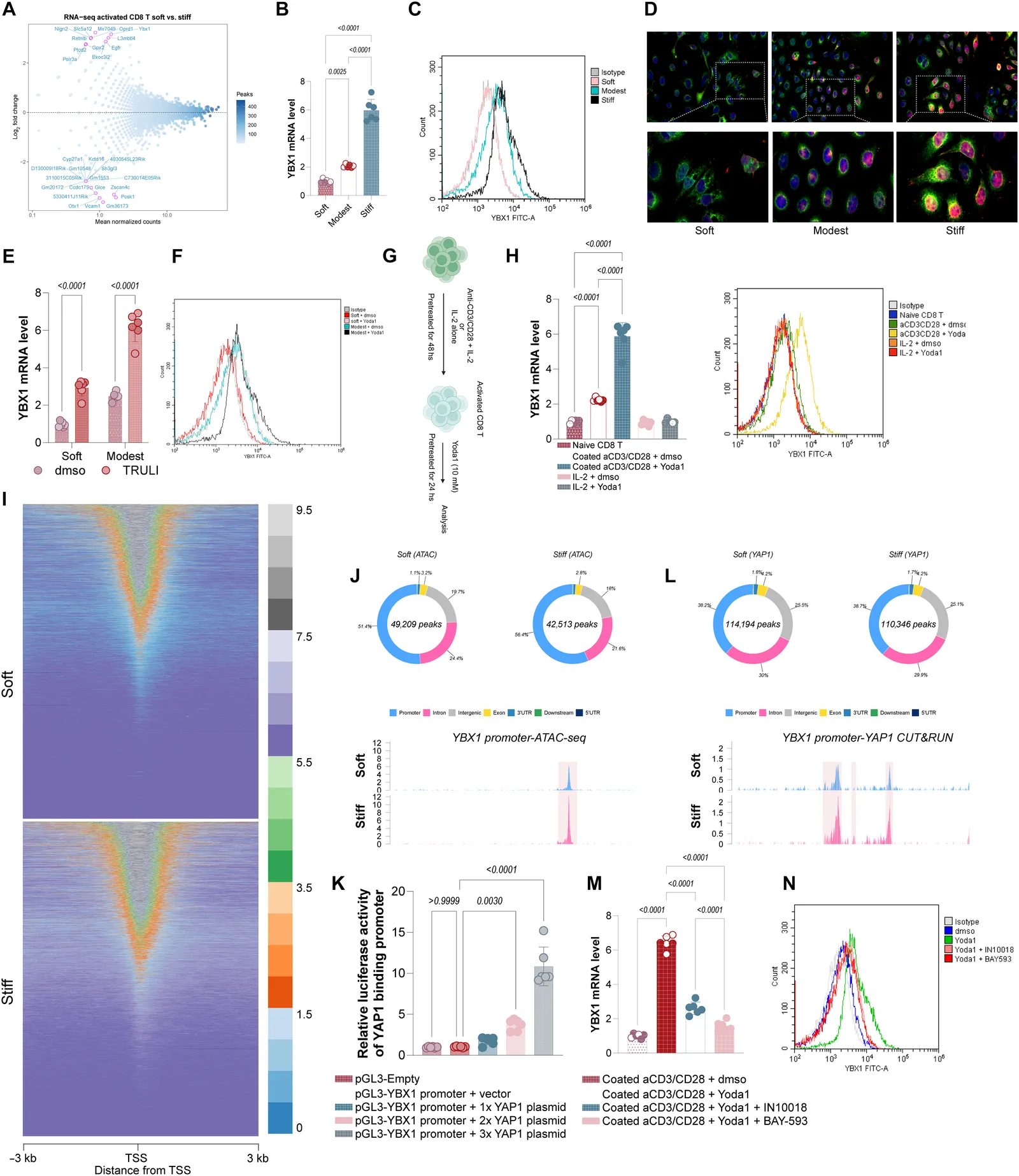
YBX1 is activated by YAP1 and participates in mechanical force–responsive CD8^+^ T cell exhaustion. (**A**) MA plots of DEGs from RNA-seq of CD8^+^ T cells seeded on soft versus stiff ECM. (**B** and **C**) qPCR and flow cytometry analysis of YBX1 expression in CD8^+^ T cells cultured in soft, moderate, or stiff gels (0, 2.5, or 5.0 μg/ml mLX4). (**D**) Nuclear translocation of YBX1 in CD8^+^ T cells cultured in soft, moderate, or stiff gels (0, 2.5, or 5.0 μg/ml mLX4) determined using immunofluorescence staining. (**E** and **F**) qPCR and flow cytometry analysis of YBX1 expression in CD8^+^ T cells cultured in soft or moderate gels (0 or 2.5 μg/ml mLX4) following TRULI treatment. (**G** and **H**) CD8^+^ T cells isolated from mice, induced with IL-2 alone or along with αCD3/αCD28 antibodies, and treated with Yoda1, followed by qPCR and FACS analysis of YBX1 expression in cells across groups. Created in BioRender. Gu, X. (2026) https://BioRender.com/tboi798. (**I**) Chromatin accessibility heatmap grouped by differential patterns in activated CD8^+^ T cells on soft (0 μg/ml mLX4) or stiff (5 μg/ml mLX4) ECM. (**J**) ATAC-seq tracks of the YBX1 locus in activated CD8^+^ T cells on soft (0 μg/ml mLX4) or stiff (5 μg/ml mLX4) ECM. (**K**) Regulation of YAP1 on YBX1 transcription determined using YBX1 promoter–driven luciferase reporter assays in 293T cells. (**L**) CUT&Tag tracks of YAP1 at the YBX1 locus in activated CD8^+^ T cells on soft (0 μg/ml mLX4) or stiff (5 μg/ml mLX4) ECM. (**M** and **N**) CD8^+^ T cells treated with Yoda1 and then FAK1 (IN10018) or YAP1 (BAY-593) inhibitors, followed by qPCR and flow cytometry analysis to analyze YBX1 expression in cells. Cell experiments repeated six times. Data are presented as dot plots and bars. Statistical significance was determined one-way ANOVA followed by Tukey’s post hoc test [(B), (H), (K), and (M)], or two-way ANOVA followed by Sidak’s post hoc test (E). Statistical significance was set at *P* < 0.05.

To delve into more molecules implicated in the CD8^+^ T cell exhaustion modulated by mechanical force, RNA sequencing (RNA-seq) analysis was used to determine differentially expressed genes (DEGs) between CD8^+^ T cells seeded on soft versus stiff gels, with YBX1 identified as a significant gene up-regulated on stiff gels (Fig. 3A. Subsequently, qPCR and flow cytometry analysis revealed increased YBX1 expression in mLX4-stimulated stiff gels (Fig. 3, B and C), but *Piezo1* knockout rendered YBX1 unresponsive to mLX4 mechanical force (fig. S7, A to C). To further investigate the spatial distribution of YBX1 in response to mechanical cues, we performed immunofluorescence staining. It was observed that stiff ECM significantly promoted the nuclear enrichment of YBX1 compared to cells on soft substrates (Fig. 3D), providing a spatial basis for its interaction with chromatin and epigenetic modifiers at exhaustion gene loci. TRULI treatment also increased YBX1 expression in CD8^+^ T cells cultured in soft or moderate gels (Fig. 3, E and F). These findings indicated that YBX1 expression is possibly increased upon mLX4-mediated Piezo activation. Furthermore, we observed that YBX1 expression was significantly up-regulated in T cell receptor (TCR) activated CD8^+^ T cells, particularly in the presence of Yoda1 treatment. However, since its expression was not markedly influenced in inactivated CD8^+^ T cells [interleukin-2 (IL-2) only] even following Yoda1 treatment (Fig. 3, G to H). Furthermore, ATAC-seq of CD8^+^ T cells on soft versus stiff ECM revealed enhanced T cell–related chromatin accessibility on stiff ECM (Fig. 3I), accompanied by an increase in YBX1 locus accessibility (Fig. 3J), consistent with its mRNA up-regulation following mechanical force stimulation. Subsequently, YBX1 promoter–driven luciferase assays demonstrated YAP1 up-regulated YBX1 in a Piezo1-dependent manner (Fig. 3K). In addition, cleavage under targets and tagmentation (CUT&Tag) assays with anti-YAP1 confirmed YAP1 binding to the YBX1 promoter under mechanical force (Fig. 3L). To clarify the regulation of FAK1-YAP1 and YBX1 via Piezo1, Yoda1 was applied to mimic mechanical force, followed by administration of FAK1 (IN10018) or YAP1 (BAY-593) inhibitors. Both inhibitors were found to attenuate Yoda1-induced YBX1 up-regulation (Fig. 3, M and N).

### YBX1 deficiency strengthens antitumor immunity of CD8^+^ T cells

**Fig. 4.**
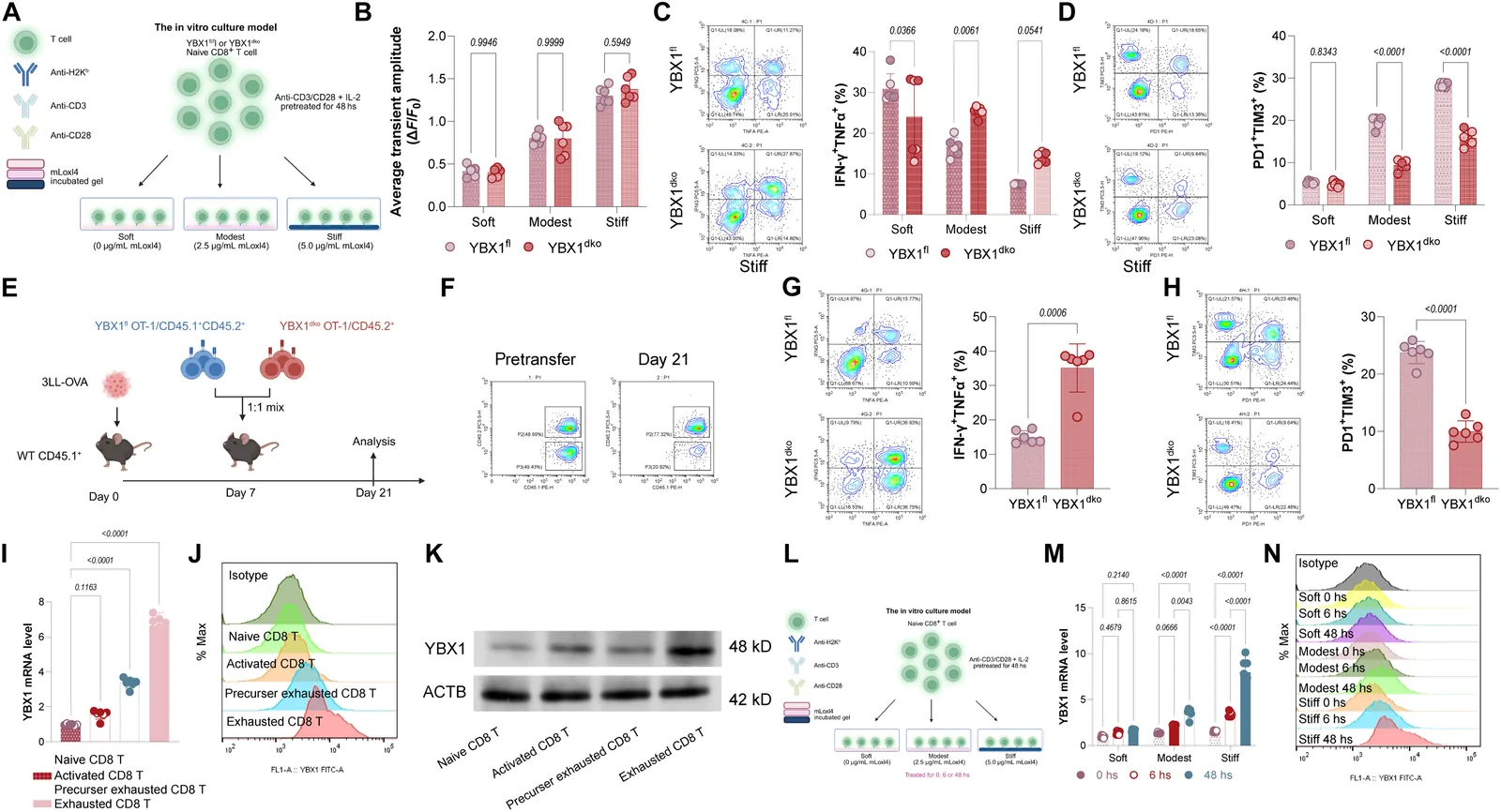
YBX1 deficiency enhances antitumor immunity of CD8^+^ T cells. (**A**) CD8^+^ T cells were extracted from *Ybx1*^fl/fl^ or *Ybx1*^dko^ mice, activated with αCD3/αCD28 antibodies, and cultured in soft, moderate, or stiff gels (0, 2.5, or 5.0 μg/ml mLX4). Created in BioRender. Gu, X. (2026) https://BioRender.com/rd5ihlz. (**B**) Quantification of average Ca^2+^ transient amplitude (Δ*F*/*F*_0_) in *Ybx1*^fl/fl^ or *Ybx1*^dko^ CD8^+^ T cells. (**C** and **D**) Populations of effector CD8^+^ T cells (TNFα^+^IFN-γ^+^) and exhausted T cells (PD-1^+^TIM-3^+^) determined using flow cytometry. (**E**) Schematic diagram of the transfer of OT1 T cells from *Ybx1*^fl/fl^ (CD45.1/CD45.2) and *Ybx1*^dko^ (CD45.2) mice (1:1 ratio) into WT mice (CD45.1) bearing 3LL-OVA tumors. Created in BioRender. Gu, X. (2026) https://BioRender.com/pwjmiqd. (**F**) Populations of CD45.1 and CD45.2 T cells in tumor tissues on day 21 determined using flow cytometry. (**G** and **H**) Populations of effector CD8^+^ T cells (TNFα^+^IFN-γ^+^) and exhausted T cells (PD-1^+^TIM-3^+^) among CD45.2^+^ cells determined using flow cytometry. (**I** to **K**) qPCR, flow cytometry, and WB analysis of YBX1 expression in naive CD8^+^ T (CD62L^+^ CD44^low^), activated CD8^+^ T (PD-1^−^TIM-3^−^), precursor exhausted T cells (PD-1^+^TIM-3^−^), and terminal exhausted T cells (PD-1^+^TIM-3^+^) isolated from lymph nodes of mice bearing 3LL tumors. (**L**) αCD3/αCD28 antibody–treated CD8^+^ T cells were seeded on 0, 2.5, or 5 μg/ml mLX4 gels for 0, 6, or 48 hours. Created in BioRender. Gu, X. (2026) https://BioRender.com/fmfj3qo. (**M** and **N**) qPCR and flow cytometry to analyze YBX1 expression in CD8^+^ T cells across groups. For animal experiments, each group contained six mice. Cell experiments repeated six times. Data are presented as dot plots and bars. Statistical significance was determined using unpaired *t* tests [(G) to (H)], one-way ANOVA followed by Tukey’s post hoc test (I), or two-way ANOVA followed by Sidak’s post hoc test [(B), (C), (D), and (M)]. Statistical significance was set at *P* < 0.05.

To further explore YBX1’s function in CD8^+^ T cell exhaustion, CD8^+^ T cells with YBX1 knockdown were generated through administration of the YBX1 short hairpin RNA (fig. S8, A and B). These cells were then activated with αCD3/αCD28 antibodies and cultured in mLX4-stimulated gels (soft, moderate, and stiff) mentioned earlier. Following YBX1 loss, Ca^2+^ influx in T cells remained unchanged (fig. S8C), although stiffness-induced CD8^+^ T exhaustion was markedly attenuated (fig. S8D-F). In addition, mice with T cell–specific *Ybx1* knockout (*Ybx1*^dko^) were generated, and their CD8^+^ T cells were extracted and seeded on soft, moderate, or stiff gels, respectively (Fig. 4A). Similarly, while no alteration in Ca^2+^ influx was observed between cells from *Ybx1*^fl/fl^ and *Ybx1*^dko^ mice (Fig. 4B), the *Ybx1*^dko^ CD8^+^ T cells showed a significant reduction in mLX4-induced exhaustion (Fig. 4, B and D). OT1 T cells from *Ybx1*^fl/fl^ (CD45.1/CD45.2) and *Ybx1*^dko^ (CD45.2) mice were adoptively transferred at 1:1 ratio into WT mice (CD45.1) bearing 3LL–ovalbumin (OVA) tumors (Fig. 4E). On day 21 posttransfer, CD45.2^+^ T cells were enriched in tumors (Fig. 4F), with higher TNFα^+^IFN-γ^+^ and lower PD-1^+^TIM-3^+^ proportions among CD45.2^+^ cells (Fig. 4, G and H). Furthermore, 3LL cells grew slower in *Ybx1*^dko^ mice compared to *Ybx1*^fl/fl^ mice (fig. S9, A and B), with enhanced effector polarization of CD8^+^ T cells and a reduction in exhaustion (fig. S9, C and D).

In addition, YBX1 expression was significantly elevated in precursor exhausted T cells (PD-1^+^TIM-3^−^), and terminal exhausted T cells (PD-1^+^TIM-3^+^) compared to naive CD8^+^ T (CD62L^+^ CD44^low^) and activated CD8^+^ T (PD-1^−^TIM-3^−^) cells isolated from lymph nodes of mice bearing 3LL tumors (Fig. 4, I to K), further suggesting the critical role of YBX1 in T cell exhaustion.

Furthermore, activated CD8^+^ T cells were seeded on soft, moderate, or stiff gels for 0, 6, or 48 hours (Fig. 4L), demonstrating a time-dependent YBX1 up-regulation on stiff gels (Fig. 4, M and N). ChIP-qPCR assays suggested the significant enrichment of YBX1 near *Pdcd1* (PD-1) and *Havcr2* (TIM-3) promoters under mechanical force (fig. S10, A and B). In addition, PD-1 and TIM-3 expression were decreased in YBX1-silenced CD8^+^ T cells (fig. S10, C and D). In another scenario, YBX1 overexpression was induced in *Piezo1*^dko^ or *Piezo1*^fl/fl^ CD8^+^ T cells, which were then cultured in stiff gels (5 μg/ml mLX4) (fig. S10E). The YBX1 overexpression promoted exhaustion of both *Piezo1*^dko^ and *Piezo1*^fl/fl^ CD8^+^ T cells. (fig. S10, F to H).

### YBX1 reduces cytotoxicity program of CD8^+^ T cells by recruiting ACLY and KAT2A

**Fig. 5.**
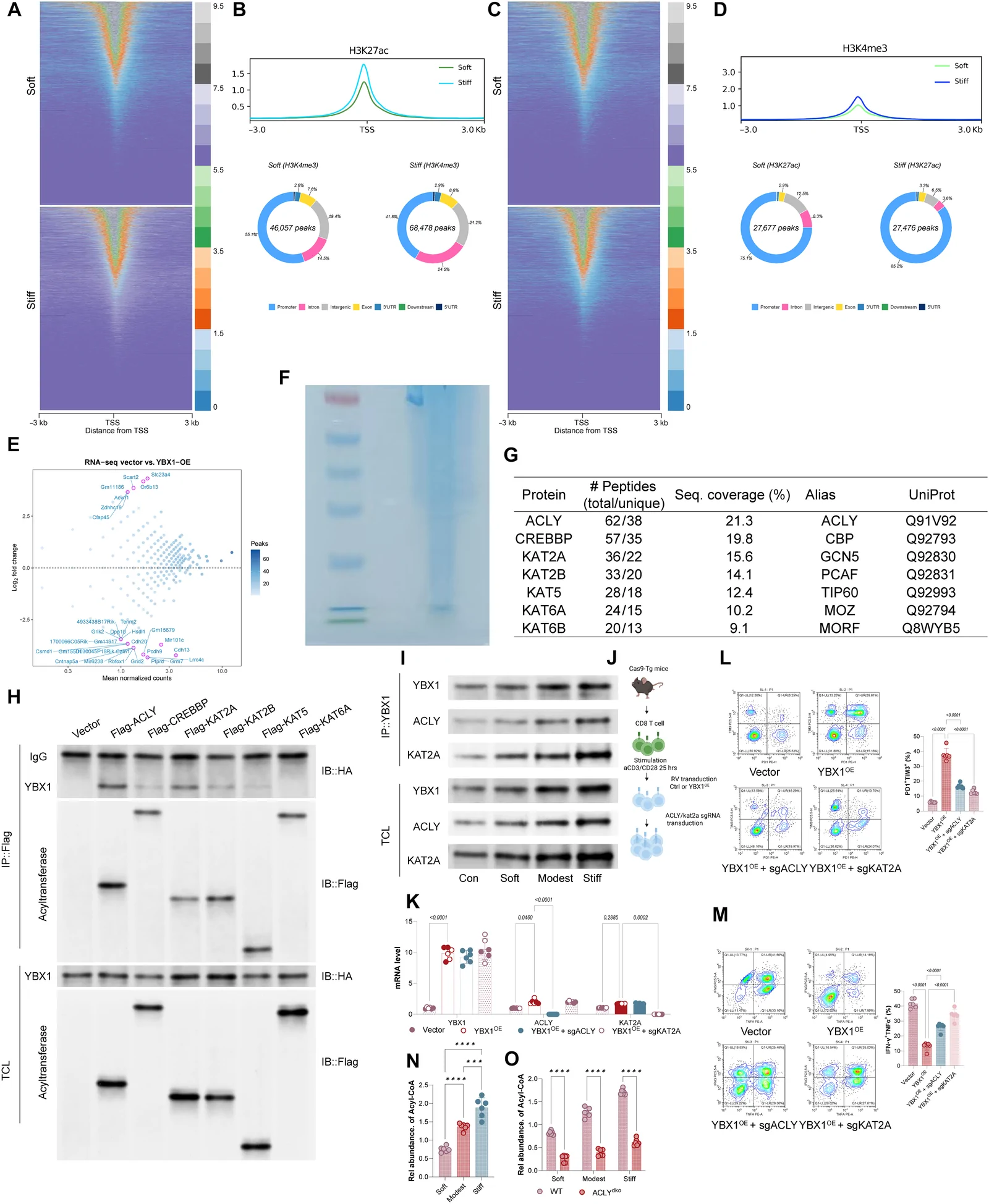
YBX1 suppresses cytotoxicity program of CD8^+^ T cells by recruiting ACLY and KAT2A. (**A** to **D**) CUT&Tag with anti-H3K27ac and anti-H3K4me3 in CD8^+^ T cells seeded on soft or stiff gels (0 or 5 μg/mL mLX4) to analyze genomic binding. (**E**) MA plots of DEGs from RNA-seq in *Piezo1*^dko^ CD8^+^ T cells overexpressing YBX1 or not. (**F** and **G**) Immunoprecipitation and mass spectrometry of YBX1-associated proteins in HEK293T cells. (**H**) Immunoblot of 293T cells expressing FLAG-tagged histone acetyltransferases and HA-YBX1, immunoprecipitated with anti-FLAG, and probed with anti-HA or anti-FLAG. (**I**) Endogenous co-IP in primary activated CD8^+^ T cells cultured on soft or stiff gels, showing the interaction between YBX1 and endogenous ACLY and KAT2A. (**J**) CD8^+^ T cells from Cas9-Tg mice transduced with YBX1 overexpression retrovirus and sgRNAs targeting *Kat2a* or *Acly*. Created in BioRender. Gu, X. (2026) https://BioRender.com/w95s9ox. (**K**) qPCR analysis of *Ybx1*, *Kat2a*, and *Acly* mRNA expression in CD8^+^ T cells following retrovirus and sgRNA administration. (**L** and **M**) Populations of effector CD8^+^ T cells (TNFα^+^IFN-γ^+^) and exhausted T cells (PD-1^+^TIM-3^+^) in CD8^+^ T cells upon YBX1, KAT2A, or ACLY alteration determined using flow cytometry. (**N** and **O**) Quantification of nuclear acetyl-CoA levels in CD8^+^ T cells cultured on soft or stiff gels (N) and in *Acly*^fl/fl^ versus *Acly*^dko^ T cells on stiff gels (O). Cell experiments repeated six times. Data are presented as dot plots and bars. Statistical significance was determined using one-way ANOVA followed by Tukey’s post hoc test [(L) to (O)], or two-way ANOVA followed by Sidak’s post hoc test (K). Statistical significance was set at *P* < 0.05.

Given the pivotal role of YBX1 in mechanical stress–mediated CD8^+^ T cell exhaustion, upstream regulation and downstream transcriptional mechanisms were explored. YBX1, a cold-shock domain family member, lacks histone modification activity but remodels chromatin by recruiting/antagonizing histone-modifying enzymes (*28*). Therefore, we hypothesized that YBX1 modulate histone modification patterns, participating in mechanical signal–induced epigenetic reprogramming to affect T cell functional states. Prior analyses showed increased CD8^+^ T cell chromatin accessibility under mechanical force. CUT&Tag with anti-H3K27ac and anti-H3K4me3 revealed elevated H3K27ac (but not H3K4me3) in CD8^+^ T cells under mechanical force (Fig. 5, A and B), primarily at promoters and distal regions (Fig. 5, C and D). RNA-seq of YBX1-overexpressing CD8^+^ T cells showed down-regulated effector-related genes and up-regulated exhaustion-related genes (Fig. 5E). To identify histone acetyltransferases interacting with YBX1, Flag-YBX1 was transfected into HEK293T cells, followed by anti-Flag IP and liquid chromatography–mass spectrometry. ACLY exhibited the strongest interaction (Fig. 5, F and G). ACLY converts citrate to acetyl–coenzyme A (CoA) for histone acetylation (*29*). Cotransfection of hemagglutinin (HA)–YBX1 with Flag-CREBBP, Flag-ACLY, Flag-KAT2A, Flag-KAT2B, Flag-KAT5, or Flag-KAT6A, followed by anti-Flag IP, revealed HA signal with Flag-KAT2A and Flag-ACLY (Fig. 5H). To confirm these interactions under physiological conditions, we performed endogenous co-immunoprecipitation (co-IP) in primary activated CD8^+^ T cells. We observed that YBX1 successfully pulled down both ACLY and KAT2A, and this interaction was markedly stronger in cells cultured on stiff gels compared to soft gels (Fig. 5I). These data indicate that YBX1 recruits ACLY and KAT2A to form a functional complex in response to mechanical force. Thus, we hypothesized that YBX1 recruits ACLY to generate acetyl-CoA, facilitating KAT2A-mediated histone acetylation. To test this, CD8^+^ T cells from Cas9-Tg mice were transduced with YBX1 overexpression retrovirus and single guide RNAs (sgRNAs) targeting *Kat2a* or *Acly* (Fig. 5, J and K). YBX1 overexpression increased the population of exhausted CD8^+^T (PD-1^+^TIM-3^+^) cells whereas it reduced the population of effector CD8^+^ T (TNFα^+^IFNγ^+^) cells, and *Kat2a* or *Acly* knockout reversed this effect (Fig. 5, L and M). Furthermore, ACLY or KAT2A overexpression vectors were introduced into *Piezo1*^dko^ CD8^+^ T cells and seeded on mLX4 gels (fig. S11, A and B). Overexpression of either ACLY or KAT2A enhanced stiffness-induced CD8^+^ T cell exhaustion (fig. S11, C to E). To confirm this metabolic link, we measured acetyl-CoA levels and found that CD8^+^ T cells cultured on stiff gels exhibited significantly higher nuclear acetyl-CoA concentrations compared to those on soft gels (Fig. 5N). This stiffness-induced increase was significantly attenuated in *Acly*^dko^ T cells, whereas cytoplasmic acetyl-CoA levels were less affected (Fig. 5O), indicating that the YBX1-ACLY complex specifically modulates the nuclear acetyl-CoA pool to facilitate localized histone acetylation at promoters at exhaustion genes (*Pdcd1* and *Havcr2*).

### ACLY or KAT2A deficiency enhances antitumor immunity of CD8^+^ T cells

**Fig. 6.**
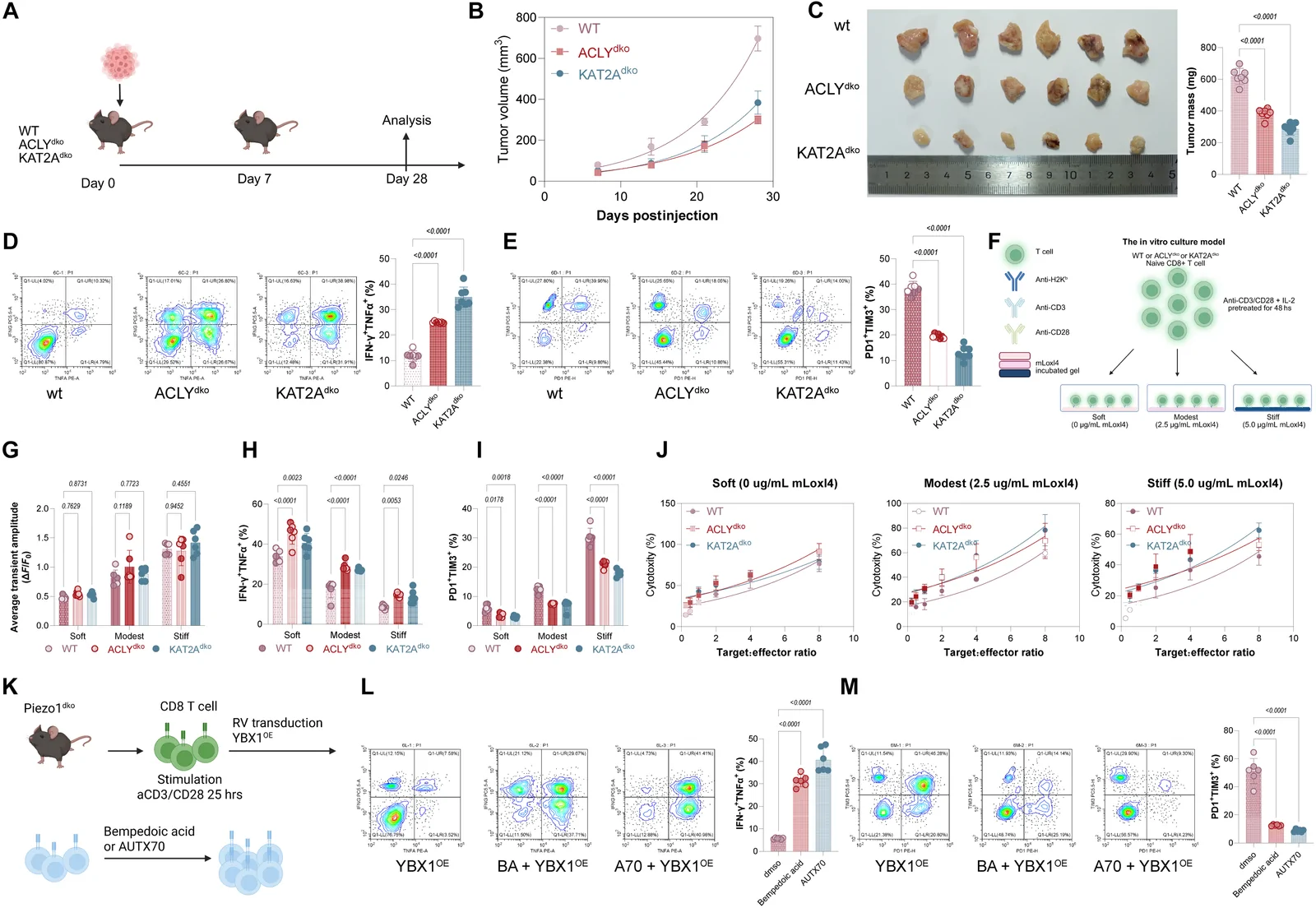
ACLY or KAT2A deficiency enhances antitumor immunity of CD8^+^ T cells. (**A**) *Acly*^fl/fl^ or *Kat2a*^fl/fl^ mice were crossed with *Cd8a*-Cre mice to generate *Acly*^dko^ or *Kat2a*^dko^ strains, followed by implantation of 3LL cells into these strains subcutaneously. Created in BioRender. Gu, X. (2026) https://BioRender.com/6ccnkis. (**B**) Weekly volume change in subcutaneous tumors in mice. (**C**) Representative images and weight of subcutaneous tumors in mice on day 28. (**D** and **E**) Populations of effector CD8^+^ T cells (TNFα^+^IFN-γ^+^) and exhausted T cells (PD-1^+^TIM-3^+^) in tumors in mice determined using flow cytometry. (**F**) CD8^+^ T cells from *Acly*^fl/fl^/*Acly*^dko^ or *Kat2a*^fl/fl^/*Kat2a*^dko^ mice were activated with αCD3/αCD28 antibodies and seeded on soft, moderate, and stiff gels (0, 2.5, or 5 μg/ml mLX4). Created in BioRender. Gu, X. (2026) https://BioRender.com/b0suxpn. (**G**) Quantification of average Ca^2+^ transient amplitude (Δ*F*/*F*_0_) in CD8^+^ T cells. (**H** and **I**) Populations of effector CD8^+^ T cells (TNFα^+^IFN-γ^+^) and exhausted T cells (PD-1^+^TIM-3^+^) determined using flow cytometry. (**J**) Cytotoxicity assay of T cells against 3LL cells. (**K**) *Piezo1*^dko^ CD8^+^ T cells overexpressing YBX1 were treated with ACLY inhibitor bempedoic acid or KAT2A inhibitor AUTX70 and seeded on 5 μg/ml mLX4 gels. Created in BioRender. Gu, X. (2026) https://BioRender.com/3upm9zi. (**L** and **M**) Populations of effector CD8^+^ T cells (TNFα^+^IFN-γ^+^) and exhausted T cells (PD-1^+^TIM-3^+^) determined using flow cytometry. Each group contained six mice. Cell experiments repeated six times. Statistical significance was determined using one-way ANOVA followed by Tukey’s post hoc test [(C), (D), (E), (L), and (M)] or two-way ANOVA followed by Sidak’s post hoc test [(B), (G) to (J)]. Data are presented as dot plots and bars. Statistical significance was set at *P* < 0.05.

To further confirm the involvement of ACLY and KAT2A in CD8^+^ T cell exhaustion, *Acly*^fl/fl^ or KAT2A^fl/fl^ mice were crossed with *Cd8a*-Cre mice to generate *Acly*^dko^ or *Kat2a*^dko^ strains, followed by implantation of 3LL cells into these strains subcutaneously (Fig. 6A). Like loss of Piezo1 or YBX1, the CD8^+^ T cell–specific *Acly* or *Kat2a* knockout in mice substantially restricted tumorigenic capacity of 3LL cells (Fig. 6, B and C) and increased effector polarization of CD8^+^ T cells (Fig. 6, D and E). Moreover, CD8^+^ T cells from *Acly*^fl/fl^/*Acly*^dko^, or *Kat2a*^fl/fl^/*Kat2a*^dko^ mice were activated with αCD3/αCD28 antibodies and seeded on soft, moderate, and stiff gels (Fig. 6F). While ACLY or KAT2A loss did not affect mLX4-induced Ca^2+^ influx in CD8^+^ T cells (Fig. 6G), the T cell exhaustion was substantially attenuated and cell cytotoxicity was increased (Fig. 6, H to J). In addition, *Piezo1*^dko^ CD8^+^ T cells overexpressing YBX1 were treated with ACLY inhibitor bempedoic acid or KAT2A inhibitor AUTX70 and seeded on 5 μg/ml mLX4 gels (Fig. 6K). Inhibitors markedly reduced YBX1-induced exhaustion (Fig. 6, L and M).

Furthermore, 3LL cells (empty vector or LOXL4-overexpressing) were implanted in C57 mice; on day 7, OT1 CD45.2^+^ cells from *Acly*^dko^ or *Kat2a*^dko^ mice were adoptively transferred (fig. S12A). Regardless of LOXL4 overexpression, transfer of *Acly*^dko^ or *Kat2a*^dko^ OT1 cells suppressed 3LL growth (fig. S12, B to E) and reduced exhausted T cells in tumors (fig. S12, F and G).

### Acetyldigoxin targets LOXL4 and suppresses lung tumorigenesis in mice

The findings above suggest the critical contribution of LOXL4 to ECM stiffness and CD8^+^ T cell dysfunction in lung cancer, suggesting it as a promising target for lung tumor progression. However, no specific drugs targeting LOXL4 are currently available. Thus, molecular docking was performed to Food and Drug Administration (FDA)–approved compounds binding LOXL4, identifying the top 6 (fig. S13).

Mouse 3LL cells were treated with these six drugs, respectively. LOXL4 mRNA remained unchanged, but protein levels were decreased, with acetyldigoxin causing the most pronounced reduction (fig. S14, A to C). Furthermore, treatment of 3LL or KLN205 cells with acetyldigoxin led to a marked increase in the ubiquitination of LOXL4 (fig. S14D). Consistently, in vitro ubiquitination assays demonstrated that acetyldigoxin treatment decreased the protein stability of LOXL4 (fig. S14E). Moreover, acetyldigoxin treatment significantly restricted proliferation and colony formation of 3LL and KLN205 cells in vitro (fig. S14, F and G). To further verify that these effects were specifically mediated via LOXL4 inhibition rather than nonspecific Na^+^/K^+^ adenosine triphosphatase (ATPase)–mediated toxicity, we treated *Loxl4*^ko^ 3LL cells with acetyldigoxin. Notably, *Loxl4*^ko^ cells exhibited significantly reduced sensitivity to acetyldigoxin compared to WT cells, confirming that LOXL4 is a primary functional target in this context (fig. S14H).

Concurrently, we established organoid models from two cases of LUAD and treated them with acetyldigoxin (fig. S14I). We observed that acetyldigoxin treatment significantly inhibited the growth of these LUAD organoids (fig. S14J).

**Fig. 7.**
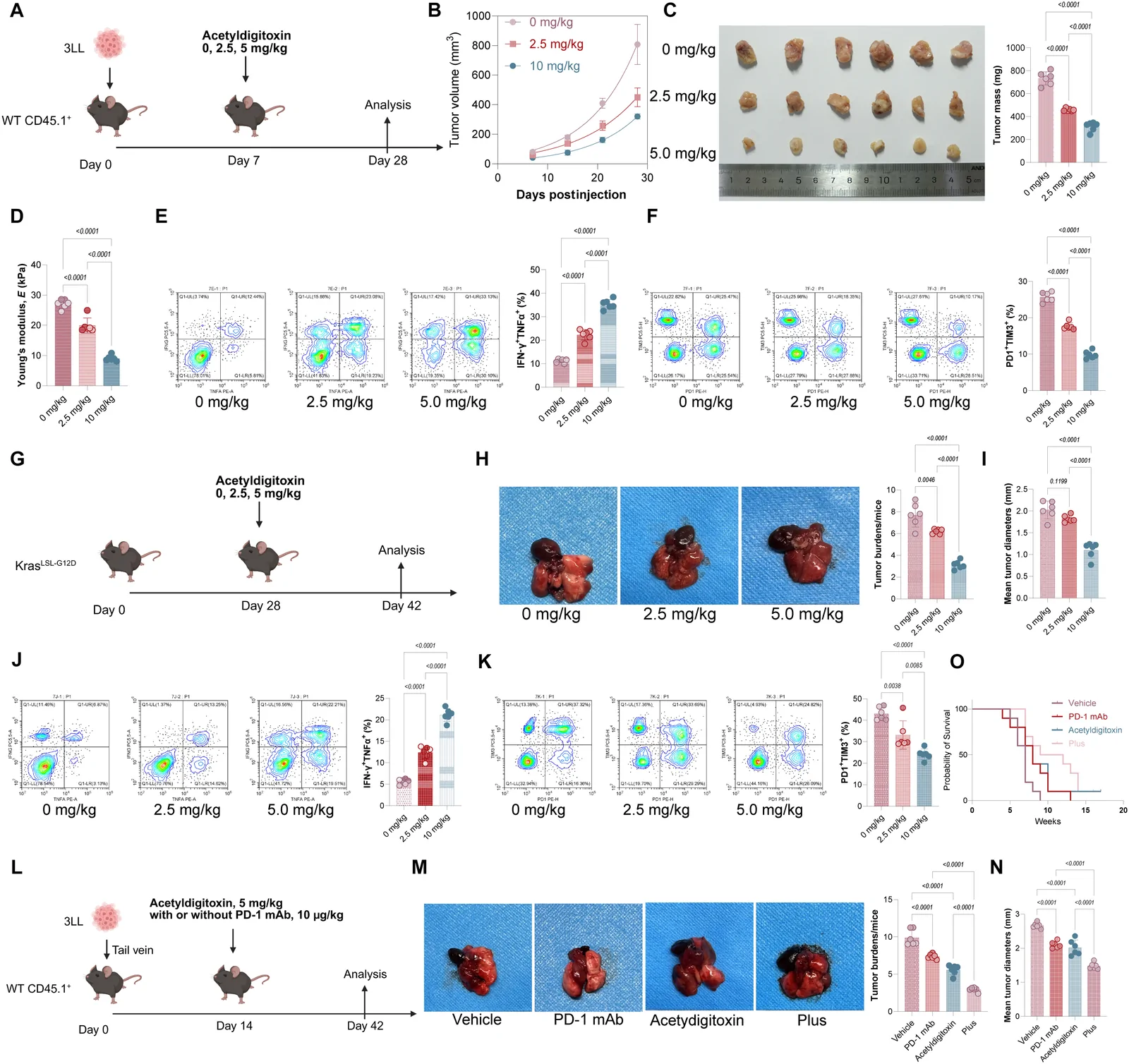
Acetyldigoxin targets LOXL4 and suppresses lung tumorigenesis in mice. (**A**) C57 mice receiving subcutaneous injection of 3LL cells were further treated with acetyldigoxin (0, 2.5, or 5 mg/kg). Created in BioRender. Gu, X. (2026) https://BioRender.com/4tfgf9i. (**B**) Weekly volume change in subcutaneous tumors formed by 3LL cells in mice. (**C**) Representative images and weight of subcutaneous tumors in mice on day 28. (**D**) Average tissue stiffness in 3LL-formed tumors evaluated by elastic modulus measurement. (**E** and **F**) Populations of effector CD8^+^ T cells (TNFα^+^IFN-γ^+^) and exhausted T cells (PD-1^+^TIM-3^+^) in subcutaneous tumor tissues determined using flow cytometry. (**G**) *Kras*^LSL-G12D^ mice were applied as a primary lung cancer model, which were administered acetyldigoxin (0, 2.5, or 5 mg/kg) from day 28. Created in BioRender. Gu, X. (2026) https://BioRender.com/862dv0c. (**H** and **I**) Gross images, tumor burdens, and mean tumor diameter in mouse lung tissues. (**J** and **K**) Populations of effector CD8^+^ T cells (TNFα^+^IFN-γ^+^) and exhausted T cells (PD-1^+^TIM-3^+^) in primary lung tumor tissues determined using flow cytometry. (**L**) C57 mice underwent tail vein injection of 3LL cells, followed by treatment of acetyldigoxin (5 mg/kg), anti–PD-1 (10 μg), or the two in combination. Created in BioRender. Gu, X. (2026) https://BioRender.com/3n7qx3x. (**M** and **N**) Gross images and mean tumor diameter of 3LL-derived metastatic nodules in mouse lung tissues. (**O**) Kaplan-Meier analysis of mouse survival (log-rank tests). Each group contained six mice. Data are presented as dot plots and bars. Statistical significance was determined using one-way ANOVA followed by Tukey’s post hoc test [(C) to (F), (H) to (K), and (M) and (N)], or two-way ANOVA followed by Sidak’s post hoc test (B). Statistical significance was set at *P* < 0.05.

In addition, C57 mice receiving subcutaneous injection of 3LL cells were further treated with acetyldigoxin (0, 2.5, or 5 mg/kg) (Fig. 7A). Acetyldigoxin dose-dependently restricted tumorigenesis of 3LL cells (Fig. 7, B and C), reduced tumor tissue stiffness (Fig. 7D), and substantially increased effector T cells while reducing exhausted T cells (Fig. 7, E and F). *Kras*^LSL-G12D^ mice were applied as a primary lung cancer model, which were administered acetyldigoxin (0, 2.5, or 5 mg/kg) from day 28 (Fig. 7G). In this model, acetyldigoxin similarly reduced tumor burden in the mouse lung tissues (Fig. 7, H and I) and increased the population of effector CD8^+^ T cells (Fig. 7, J and K). In contrast, the 2.5 mg/kg dose group showed only a marginal reduction in these metrics, indicating a threshold effect where a higher dose is required to achieve meaningful therapeutic outcomes. This dose dependency aligns with the requirement for potent LOXL4 inhibition to disrupt the dense, cross-linked collagen network and restore CD8^+^ T cell infiltration.

To assess the application potential of acetyldigoxin in lung cancer therapy, acetyldigoxin (5 mg/kg), or anti–PD-1 (10 μg/kg), or the two in combination, was administered in mice underwent tail vein injection of 3LL cells (Fig. 7L). Acetyldigoxin showed synergistic effects with anti–PD-1 in reducing tumor metastasis and growth (Fig. 7, M and N) and prolonging mouse survival (Fig. 7O).

## DISCUSSION

Immunotherapy now stands as a foundational pillar in oncology, with especially transformative impact in the management of lung cancer (*3*). Yet, many patients fail to benefit from ICB, partly because of an immune-excluded phenotype in which effector T cells are trapped in dense peritumoral stroma (*30*–*32*). Structural and compositional anomalies within the ECM impose a biomechanical impediment to T cell trafficking and interstitial migration (*33*). In this study, we report that LOXL4-driven ECM stiffening induces CD8^+^ T cell exhaustion via Piezo1-mediated mechanosignaling. Targeting LOXL4 shows promising synergistic effects with ICB in lung cancer management.

While emerging studies have concerned the function of LOXL4 in human malignancies, reports on its role in lung cancer remain controversial. Chen and colleagues (*34*) suggested that LOXL4, poorly expressed in lung cancer tissues and cell lines, activated ECM and augmented proliferation and mobility of human lung cancer cells. By contrast, loss of LOXL4 upon microRNA-328-5p targeting has been observed to enhance lung tumorigenesis (*35*). We observed that *Loxl4* knockout retarded subcutaneous tumor growth of 3LL cells in mice, with more pronounced suppression in immunocompetent mice over that in immunodeficient ones. This indicates an intrinsic protumorigenic role for LOXL4 in lung cancer, with these effects amplified by immune interactions in competent hosts. LOXL4 has been found to be up-regulated and correlated with the expression of immune checkpoint molecules across multiple cancers (*36*). During hepatocarcinogenesis, increased LOXL4 expression has been found to invoke the immunosuppressive, adaptive skewing of tumor-associated macrophages and activate PD-L1 expression (*37*). Similarly, Zhao *et al.* (*38*) demonstrated that tumor cells–derived exosomal LOXL4 activates STAT1/PD-L1 in macrophages, inhibiting CD8^+^ killing and promoting exhaustion in hepatocellular carcinoma. Here, we found that *Loxl4* knockout in 3LL cells led to increased effector CD8^+^ T cells (TNFα^+^IFN-γ^+^) and reduced exhausted T cells (PD-1^+^TIM-3^+^) in the formed subcutaneous tumors. By contrast, in vitro, mLX4 supplementation in collagen-formed gels dose-dependently impaired CD8^+^ T cell function and cytotoxicity. Increased ECM stiffness coupled with compact collagen architectures can physically obstruct the entry and movement of immune effectors, thereby curtailing direct cytotoxic engagement with malignant targets (*17*, *39*). Beyond mere barriers to trafficking, matrix stiffness exerts profound regulatory influence over T cell biology, encompassing clonal expansion, lineage commitment, and effector potency (*40*). Softer, less rigid substrates have been shown to promote the outgrowth of both CD4^+^ and CD8^+^ T lymphocytes, alongside augmented production of IL-2 (*41*, *42*). Furthermore, optimal T cell activation and cytotoxic function require enhanced metabolic flux and nutrient acquisition; however, stiffened ECM can sequester critical nutrients, thereby compromising effector differentiation and anti-tumor activity (*43*). Elevated intratumoral collagen density is associated with diminished CD8^+^ T cell infiltration, an elevated CD4^+^/CD8^+^ ratio, and down-regulated expression of granzyme and perforin, collectively indicative of impaired cytotoxic capacity (*41*, *44*). Collectively, these insights and findings from our laboratory suggests a direct effect of LOXL4-driven collagen cross-linking on lung cancer progression through ECM stiffness–favored proliferation and metastasis of tumor cells as well as exhaustion of CD8^+^ T cells.

Further investigations in downstream effectors of LOXL4 showed that stiff ECM (via mLX4) increases Ca^2+^ influx in CD8^+^ T cells; Piezo inhibitor (GsMTx4) blocks exhaustion, while TRPV4 inhibitor (GSK205) did not. Piezo1, a mechanosensitive calcium-permeable channel characterized by rapid adaptation, is abundantly expressed in tissues subjected to recurrent mechanical forces, such as skin, blood vessels, and various immune subsets (*45*). It serves as the predominant mechanotransducing Ca^2+^ conduit in cells exposed to heightened matrix rigidity (*46*). Notably, we observed that the growth of 3LL cells was substantially restricted in *Piezo1*^dko^ compared to *Piezo1*^fl/fl^ mice, accompanied by reduced exhausted CD8^+^ T cells. In vitro, T cells from *Piezo1*^dko^ mice also exhibited minimal response to ECM stiffness. Although prior studies have established Piezo1’s facilitative role in early T cell priming and activation (like CD4^+^ T cells) during tumor challenge (*47*), within a rigid TME, Piezo1 detects mechanical stress and actively drives CD8^+^ T cell exhaustion (*24*, *48*). Additional reports indicate that Piezo1-dependent stiffness signaling promotes the differentiation of regulatory T cells, thereby reinforcing an immunosuppressive milieu (*49*). Furthermore, we observed that mLX4 treatment markedly promoted CD8^+^ T cell exhaustion in *Piezo1*^fl/fl^ mice but failed to significantly alter the populations of exhausted CD8^+^ T cells in *Piezo1*^dko^ mice, indicating the exclusive involvement of Piezo1 in LOXL4-mediated CD8^+^ T cell dysfunction.

Increased Piezo1 expression was detected CD8^+^ T cells cultured in stiff gels, accompanied by increased FAK^Tyr397^ phosphorylation and nuclear YAP1 localization. These effects were absent in *Piezo1*^dko^ CD8^+^ T cells. These observations indicate that FAK and YAP may function as critical Piezo1 effectors in stiff TME. Previous evidence has also suggested that Piezo1 triggers Ca^2+^ influx that promotes FAK autophosphorylation at Tyr^397^, which activates actin polymerization and YAP nuclear translocation (*50*). FAK phosphorylation has been found to be enhanced and play a critical role in LOX- catalyzed collagen cross-linking (*40*). Similarly, YAP1 functions as a mechanosensor in stiffened lymphoid tissues, where it suppresses T cell expansion by blocking NFAT1 nuclear entry (*51*). Both FAK and YAP pathway signaling has been found to impair CD8^+^ T cell function in the TME (*52*, *53*). YAP integrates diverse biophysical inputs, including fluid shear, cellular geometry, and ECM rigidity, translating them into lineage-specific gene expression profiles (*54*). Activated YAP1 in the nucleus facilitates transcription of various genes implicated in tumor progression (*55*). Nevertheless, our ChIP-qPCR and dual-luciferase reporter experiments revealed no direct binding of YAP1 to promoters of T cell effector or exhaustion signature genes, suggesting that additional intermediary effectors propagate the exhaustion phenotype downstream of YAP1 activation.

Further mechanistic dissection using RNA-seq, ATAC-seq, CUT&Tag, ChIP-qPCR, and luciferase reporter assays uncovered that YBX1, which was markedly induced in CD8^+^ T cells cultured on rigid substrates, is a direct transcriptional target of YAP1. Stiff ECM promoted nuclear enrichment of YBX1, providing a spatial basis for its interaction with chromatin and epigenetic modifiers at exhaustion gene loci. Increased YBX1, in turn, recruited ACLY and KAT2A to the promoter regions of *Pdcd1* (PD-1) and *Havcr2* (TIM-3), leading to increased chromatin accessibility, and activating expression of these exhaustion genes through epigenetic mechanisms. Endogenous co-IP in primary activated CD8^+^ T cells confirmed that YBX1 interacts with both ACLY and KAT2A, with markedly stronger interaction on stiff gels compared to soft gels. Although YBX1 is primarily known as a cold-shock domain RNA binding protein, its nucleic acid–binding versatility enables recognition of Y-box sequences or single-stranded DNA intermediates within promoters (*56*). In this axis, YBX1 operates as a chromatin-tethered RNA binding scaffold that coordinates multiprotein epigenetic complexes (*57*, *58*). ACLY catalyzes the conversion of citrate to acetyl-CoA, providing the substrate for histone acetylation (*59*, *60*), while KAT2A (GCN5) functions as a histone acetyltransferase that specifically relaxes chromatin through H3K9 and H3K27 acetylation (*61*, *62*). Stiff gels induced higher nuclear acetyl-CoA concentrations in CD8^+^ T cells, an increase that was attenuated in *Acly*^dko^ T cells while cytoplasmic acetyl-CoA levels were less affected, indicating that the YBX1-ACLY complex modulates the nuclear acetyl-CoA pool to facilitate localized histone acetylation at exhaustion gene promoters. A recent study by Ma and colleagues (*63*) demonstrated that YBX up-regulated neutrophil-recruiting chemokines CXCL1/CXCL3, fostering immunosuppression in gastric tumors. Similarly, KAT2A has been implicated in transcriptional induction of transforming growth factor–β, which suppresses CD8^+^ T cell effector function in hepatocellular carcinoma (*64*). Partly consistent with these insights, we observed that CD8^+^ T cell–specific knockout of YBX1, ACLY, or KAT2A in mice restricted tumorigenic capacity of 3LL cells. In vitro, genetic disruption of any of these factors left mLX4-triggered Ca^2+^ influx intact but profoundly mitigated exhaustion phenotypes and restored cytotoxic potency in CD8^+^ T cells. These findings suggest a mechanosignaling-epigenetic cascade: the Piezo1-FAK1-YAP1-YBX1-ACLY/KAT2A axis that contribute to CD8^+^ T cell dysfunction in response to LOXL4-mediated ECM stiffness.

Building upon these insights, pharmacological inhibition of LOXL4 emerges as an attractive therapeutic avenue to curb disease progression and enhance clinical responses. Currently, however, no LOXL4-specific inhibitors are clinically available. To address this gap, we conducted in silico molecular docking screens of FDA-approved agents against the LOXL4 catalytic domain, identifying acetyldigoxin as a top-ranking binder with high predicted affinity. Acetyldigoxin is a synthetic derivative of digoxin, a well-known cardiac glycoside that has traditionally been used to treat heart failure and atrial fibrillation (*65*, *66*). Digoxin and its derivatives, like acetyldigoxin, exert their effects by inhibiting the Na^+^/K^+^ ATPase pump, an essential enzyme in cell membranes that maintains the proper balance of sodium and potassium ions (*67*). Cancer cells often overexpress Na^+^/K^+^-ATPase isoforms (particularly α3 and α1), making them more sensitive to inhibition than normal cells (*68*). Beyond their cardiovascular properties, digoxin and its derivatives have shown potentials in cancer management. For instance, in a study isolating cardiac glycosides from *Digitalis lanata*, acetyldigoxin exhibited strong in vitro cytotoxicity against HeLa (cervical cancer) cells, A549 (NSCLC) cells, and MCF-7 (breast cancer) cells (*69*). A recent report by Kurzeder *et al.* (*70*) suggested that digoxin reduced circulating tumor cell clusters in metastatic breast cancer. More relevantly, digoxin has been demonstrated to sensitize NSCLC cells to chemotherapy through Na^+^/K^+^-ATPase inhibition (*71*). However, the influence of digoxin or its derivatives in tumor immunity remains elusive. In this study, we observed that acetyldigoxin markedly reduced LOXL4 protein levels in 3LL cells without altering LOXL4 mRNA expression, suggesting posttranscriptional regulation (either direct or indirect). Acetyldigoxin treatment increased ubiquitination of LOXL4 and decreased its protein stability. Loxl4ko 3LL cells exhibited significantly reduced sensitivity to acetyldigoxin compared to WT cells, confirming that LOXL4 is the primary functional target. Moreover, acetyldigoxin dose-dependently restricted tumorigenesis of 3LL cells in mice and suppressed primary lung tumorigenesis in *Kras*^LSL-G12D^ mice, accompanied by an increase in effector CD8^+^ T cells. The observed threshold effect at 2.5 mg/kg (with only marginal benefits in primary tumor metrics) underscores the need for sufficient drug exposure to overcome fibrotic stroma; future pharmacokinetic studies are warranted to optimize dosing and widen the therapeutic window while minimizing potential cardiac glycoside–related toxicities. In a patient-derived organoid model, acetyldigoxin treatment also reduced the LUAD-organoid development in vitro. Furthermore, acetyldigoxin showed synergistic effects with anti–PD-1 in reducing tumor metastasis and growth in mice receiving tail vein injection of 3LL cells. Collectively, these findings position acetyldigoxin as a repurposable candidate with dual antitumor and immune-potentiating properties in lung cancer.

Several limitations should be acknowledged. First, although molecular docking predicted high-affinity binding of acetyldigoxin to LOXL4 and we observed potent reduction of LOXL4 protein, the precise binding site, on-target specificity, and exact mechanism of LOXL4 degradation remain to be elucidated. Second, while LOXL4 overexpression correlated with reduced CD8^+^ T cell infiltration in human TMA and TCGA datasets, future studies should examine whether the LOXL4-Piezo1-YBX1 axis and response to LOXL4 inhibition differ across histological subtypes of lung cancer, including adenocarcinoma versus squamous cell carcinoma and NSCLC versus small cell lung cancer. When applicable, larger clinical cohorts are required to definitively establish the prognostic significance of LOXL4 and downstream effectors (Piezo1, YBX1, ACLY, and KAT2A) in patients with lung cancer.

**Fig. 8.**
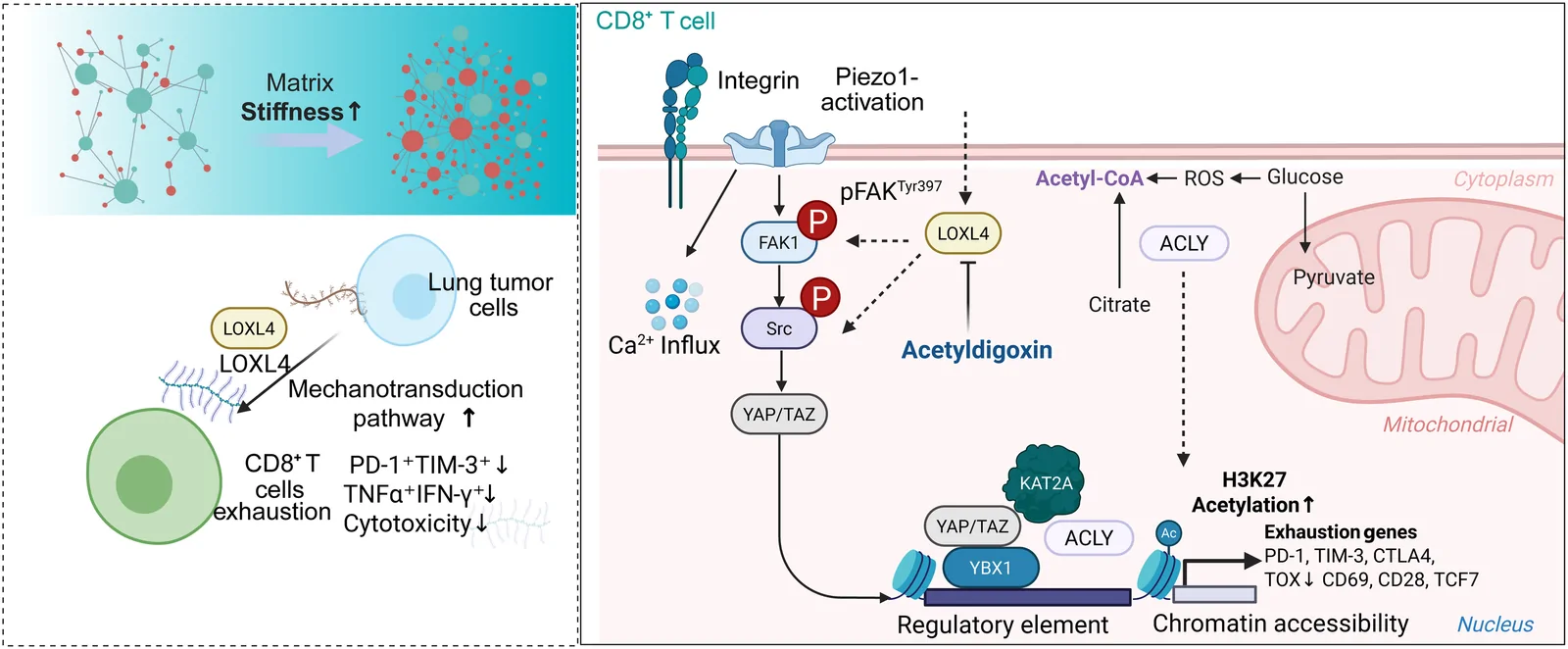
Graphical abstract. This study uncovers a previously unknown mechanotransduction-to-epigenetic pathway by which LOXL4-driven matrix stiffening induces CD8^+^ T cell exhaustion. Repurposing of the cardiac glycoside derivative acetyldigoxin as a LOXL4-targeted therapy offers a clinically translatable strategy to overcome ECM-mediated immunotherapy resistance in lung cancer. Created in BioRender. Gu, X. (2026) https://BioRender.com/ge5v729.

In conclusion, our work elucidates a mechanistic cascade whereby LOXL4 reinforces ECM rigidity, which in turn engages Piezo1 to trigger FAK-YAP1 signaling, culminating in YBX1-facilitated recruitment of ACLY and KAT2A to exhaustion gene loci, thereby driving CD8^+^ T cell dysfunction. These findings provide previously unidentified evidence of the supportive role of collagen cross-linking/ECM stiffness in tumor growth and CD8^+^ T dysfunction. Critically, we identify acetyldigoxin as a potential high-affinity LOXL4 inhibitor with robust antitumor efficacy across multiple preclinical lung cancer models without causing significant organ damage or other adverse effects on the experimental hosts. Our findings unveil LOXL4-driven mechano-epigenetic regulation of T cell exhaustion and nominate acetyldigoxin as a promising therapeutic agent to overcome ECM-mediated immunotherapy resistance (Fig. 8).

## MATERIALS AND METHODS

### Ethics approval and consent to participate

This study and included experimental procedures were approved by the institutional animal care and use committee of Shanghai Pulmonary Hospital, School of Medicine, Tongji University (approval number: K25-392Y). All animal housing and experiments were conducted in strict accordance with the institutional guidelines for the care and use of laboratory animals.

### Immunohistochemistry

Immunohistochemistry was performed on a TMA containing 117 pairs of LUAD and adjacent nontumor tissues to evaluate protein expression. Formalin-fixed, paraffin-embedded tissue sections were deparaffinized in xylene and rehydrated through a graded ethanol series, followed by antigen retrieval in citrate buffer at pH 6.0 under high heat. After blocking endogenous peroxidase activity with hydrogen peroxide and nonspecific binding with goat serum, the sections were incubated overnight at 4°C with primary antibodies targeting LOXL4, CD8, and GZMB. Subsequently, the slides were washed and incubated with a horseradish peroxidase (HRP)–conjugated secondary antibody, visualized using a 3,3′-diaminobenzidine substrate system, and counterstained with hematoxylin. Staining intensity was quantified by two independent pathologists using the H-score method, which multiplies the percentage of positive cells by the staining intensity (0 to 3), yielding a score range of 0 to 300 to stratify patients into high and low expression groups based on the median value. To validate the consistency of the observations, inter-rater reliability was assessed using the intraclass correlation coefficient (ICC), which demonstrated excellent agreement between the two pathologists (ICC = 0.92)

### Establishment and culture of patient-derived organoids

LUAD organoids were established by washing fresh tumor tissues with cold phosphate-buffered saline (PBS) containing antibiotics, followed by mechanical mincing and enzymatic digestion with collagenase and hyaluronidase at 37°C. The resulting cell suspension was filtered and centrifuged, and the pellet was resuspended in growth factor–reduced Matrigel and seeded into prewarmed culture plates to form domes. After the matrix solidified, the organoids were overlaid and cultured in human lung organoid medium supplemented with B27 (1×), *N*-acetylcysteine (1.25 mM), epidermal growth factor (50 ng/ml), Noggin (100 ng/ml), and R-spondin 1 (500 ng/ml). The cultures were maintained in a humidified incubator at 37°C, and the medium was replenished every 3 days to ensure the continuous supply of growth factors and nutrients.

### Cell viability and drug treatment assays

To assess the therapeutic efficacy of acetyldigoxin, organoids were dissociated into single cells using TrypLE Express and reseeded in Matrigel within white-walled 96-well plates. Following a recovery period, the organoids were treated with acetyldigoxin (MedChemExpress, #HY-121573) at indicated concentrations for durations of 0, 3, 7, and 14 days, with the drug-containing medium refreshed every 3 days. Cell viability was subsequently quantified using the CellTiter-Glo Luminescent Cell Viability Assay, where the reagent was added to the culture wells, shaken to induce cell lysis, and incubated at room temperature to stabilize the signal before measuring luminescence on a microplate reader to calculate relative growth inhibition.

### TCGA database and immune infiltration analysis

Transcriptomic data and corresponding clinical information for the TCGA-LUAD and TCGA-LUSC cohorts were obtained from the UCSC Xena browser to investigate the immunological landscape of lung cancer. The relative proportions of 22 distinct tumor-infiltrating immune cell types were estimated for each sample using the CIBERSORT deconvolution algorithm with the LM22 signature matrix and 1000 permutations. Samples with a CIBERSORT *P* value less than 0.05 were retained for downstream analysis, and the association between *LOXL4* expression levels and the infiltration abundance of CD8-positive T cells and cytotoxic T lymphocytes was evaluated using Pearson correlation coefficients.

### Animals

All animal experiments were approved by the Institutional Animal Experiment Ethics Committee of Shanghai Pulmonary Hospital, School of Medicine, Tongji University (approval number: K25-392Y). Mice used in the experiments included C57BL/6J (C57), BALB/c nude mice (female, 6 to 8 weeks old, specific pathogen–free (SPF) grade, purchased from Anburui BioTechnology Co. Ltd., Fujian, China), *Piezo1*^flox/flox^, *Piezo1^dko^*, *Ybx1^fl/fl^*, *Acly*^fl/fl^, *Kat2a*^fl/fl^, and *Cd8a*-Cre mice (the Jackson Laboratory, Bar Harbor, USA). Mice of each strain were maintained in an SPF-grade animal facility with environmental temperature maintained at 22° ± 2°C, 55 ± 5% humidity, a 12-hour light/dark cycle, and unlimited access to autoclaved standard pellet feed and drinking water. Piezo1, *Ybx1*, *Acly*, and *Kat2a* T cell–conditional knockout mouse lines were obtained by crossing *Cd8a*-Cre mice with respective flox/flox mice, which were designated as *Piezo1^dko^*, *Ybx1*^dko^, *Acly*^dko^, and *Kat2a*^dko^ strains, respectively. Genotyping was performed via tail-tip DNA extraction (Proteinase K digestion, 55°C overnight) followed by PCR identification. Random number tables were used for group allocation and blinded analysis in all animal experiments to ensure objectivity and reproducibility. Unless otherwise specified, each group consisted of six mice. When tumor burden exceeded 1200 mm^3^ or animals exhibited overt pain or distress, isoflurane anesthesia was immediately administered followed by euthanasia to minimize suffering.

### Cell culture

Mouse Lewis lung carcinoma cell line 3LL (CRL-1642) and squamous lung carcinoma cell line KLN205 (CCL-216) were acquired from American Type Culture Collection (ATCC, Manassas, VA, USA), authenticated by STR profiling, and verified for species origin before use. Cells were cultured in high-glucose Dulbecco’s modified Eagle’s medium (Gibco, #11995073) containing 10% fetal bovine serum (FBS, Gibco, #10099141C) and 1% antibiotic mixture (Gibco, #15140122), and maintained in a 37°C, 5% CO_2_, saturated humidity incubator (Thermo Fisher Scientific Series 8000). Cells at 80 to 90% confluence were passaged using 0.25% trypsin-EDTA (Gibco, #25200056) at a 1:3 to 1:5 ratio, with medium refreshed every 3 days. Only cells with passage number ≤10 were used to ensure stability. Primary mouse CD8^+^ T cells were isolated from spleen tissue by mechanical grinding into single-cell suspensions, followed by purification using CD8α MicroBeads (Miltenyi, #130-117-044) on the MACS magnetic separation system, achieving purity >95%. Purified cells were seeded in RPMI 1640 medium (Gibco, #11875093) containing 10% FBS, 50 μM β-mercaptoethanol (Sigma-Aldrich, #M6250), and IL-2 (20 U/ml; PeproTech #212-12), and activated for 24 hours on plates precoated with αCD3/αCD28 antibodies (2 μg/ml each) before downstream experiments. The purity of the isolated cells was verified by flow cytometry (CD3^+^CD8^+^) for each independent preparation, consistently achieving a purity of >95% before use in downstream experiments.

### Tumor cell implantation

Mouse lung cancer models were established using Lewis lung carcinoma 3LL cells or 3LL-OVA cells expressing OVA antigen. Exponentially growing cells were digested and resuspended in PBS to 1 × 10^7^ cells/ml. Each mouse received a subcutaneous implantation of 1 × 10^6^ cells (100 μl PBS) into the right dorsal subcutaneous fat layer using a 27G needle, avoiding skin penetration to prevent bubble formation. Postimplantation, mice were monitored daily for mental status, food intake, and body weight. Tumor volume was measured every 7 days using a vernier caliper to record length (L) and width (W), with volume calculated using the formula *V* = (*L* × *W*^2^) × π/6. After 28 days, or when tumor volume exceeded 1200 mm^3^ or humane end points were met (e.g., impaired mobility, >20% body weight loss), mice were then anesthetized with isoflurane and euthanized.

### Pharmacologic treatment and grouping

When subcutaneous tumor volume reached approximately 50 to 80 mm^3^ (day 7 postimplantation), mice were randomly allocated into groups for distinct treatments. mLX4 [purity ≥95% by SDS–polyacrylamide gel electrophoresis (SDS-PAGE); KMD Bioscience] was dissolved in PBS and administered intratumorally at 2.5 or 5 μg per tumor every 3 days for three doses. Acetyldigoxin was dissolved in 0.5% methylcellulose solution and intraperitoneally administered at 2.5 or 5 mg/kg daily for 10 consecutive days; control groups received an equivalent volume of vehicle. TRULI (MCE, #HY-138489), a potent LATS1/2 inhibitor, was dissolved and administered intratumorally at 1 mg/kg daily for 10 days. In immune combination experiments, mouse monoclonal antibody against PD-1 (BioXcell, #BE0273) was administered intraperitoneally at 10 μg/kg starting on day 7 postimplantation, every 3 days for three doses. During treatment, all drug doses were adjusted on the basis of body weight and administered within the same time window, with tumor growth, body weight, and general condition monitored.

### Adoptive transfer of T cells

Spleens and lymph nodes were harvested from OT-1 mice (CD45.1/CD45.2) and *Ybx1*^dko^ OT-1 mice (CD45.2) following cervical dislocation. CD8^+^ T cells were purified using CD8α magnetic beads, with purity verified by flow cytometry for each replicate to ensure that it exceeded 95%. Purified cells were cultured for 24 hours in 24-well plates coated with αCD3/αCD28 antibodies (2 μg/ml each; Invitrogen, #11456D) with IL-2 stimulation (20 U/ml) to activate and maintain viability. Activated CD45.1/CD45.2 OT-1 and *Ybx1*^dko^ OT-1 T cells were mixed at a 1:1 ratio (total 1 × 10^7^ cells per mouse) and injected via tail vein into CD45.1 mice bearing 3LL-OVA tumors for 7 days. On day 21, tumors, spleens, and peripheral blood were collected for flow cytometry to analyze CD45.1 and CD45.2 T cell ratios and effector/exhaustion phenotypes.

### AFM of tumor stiffness

Tumor tissue was excised immediately after animal euthanasia, rinsed in ice-cold PBS, and maintained in a hydrated state for mechanical stiffness measurement on an atomic force microscopy (AFM) platform (Bruker NanoWizard III). Measurements were performed using a 2-μm radius spherical probe (spring constant 0.06 N/m) at a constant temperature of 25°C with an indentation rate of 2 μm/s for force curve acquisition. Five independent regions were randomly selected per sample, with 20 points measured per region. Force-displacement curves were fitted using the Hertz model to determine Young’s modulus (in kilopascal), reflecting ECM stiffness. To prevent tissue dehydration, measurements were conducted under PBS coverage throughout. Raw data were analyzed using JPK Data Processing software (v6.1) to compute mean values, with at least six independent samples included per group.

### Collagen gel preparation and stiffness measurement

All procedures were performed on ice to prevent premature collagen polymerization. Type I collagen stock solution (Corning, #354236, 3 mg/ml) was neutralized to pH 7.4 by mixing with sterile 10× PBS, 0.1 N NaOH, and sterile distilled water to prepare a final collagen solution (2 mg/ml). High-purity (>95%) mLX4 was added to final concentrations of 0, 2.5, or 5 μg/ml, gently mixed, and immediately dispensed into 24-well plates (300 μl per well) or glass-bottom dishes. Gels were allowed to polymerize fully at 37°C for 1 hour and then overlaid with complete RPMI 1640 medium and equilibrated at 23° to 25°C for 30 min. Gel mechanical properties were assessed by AFM with measurement procedures described above.

### CD8^+^ T cell seeding and stimulation

Naïve CD8^+^ T cells were extracted from C57BL/6J mouse spleens as single-cell suspensions and purified using CD8α MicroBeads. Purified cells were resuspended in RPMI 1640 medium and seeded onto collagen gels of varying stiffness (treated with 0, 2.5, or 5 μg/ml mLX4) at 2 × 10^5^ cells per well. Gel surfaces were precoated with αCD3/αCD28 antibodies (2 μg/ml each) to activate TCR signaling. Cells were cultured at 37°C, 5% CO_2_ for 48 hours, then harvested, washed, and analyzed by flow cytometry to assess changes in TNFα^+^IFN-γ^+^ effector T cells and PD-1^+^TIM-3^+^ exhausted T cells. IL-2 was refreshed every 24 hours to sustain T cell activation.

### Measurement of intracellular Ca^2+^ influx

To evaluate the impact of ECM stiffness and Piezo channel activation on CD8^+^ T cell calcium signaling, purified CD8^+^ T cells were stimulated with αCD3/αCD28 antibodies for 24 hours and then loaded with 2 μM Fluo-4 AM calcium probe (Invitrogen, #F14201) in Hanks’ balanced salt solution (HBSS) buffer (containing 1 mM CaCl_2_ and 1 mM MgCl_2_) at 37°C for 30 min with gentle agitation. After loading, cells were washed three times with HBSS to remove extracellular probe and rested for 10 min in 5% CO_2_ to allow probe esterification. Real-time calcium signals were acquired on a Zeiss LSM 880 confocal microscope (excitation 488 nm, emission 520 nm, 63× oil numerical aperture 1.4 objective, 1 frame/s, 300 s duration). Representative kinetic traces were generated by plotting the mean Δ*F*/*F*_0_ values from at least 20 individual cells per condition over the 300-s recording period to visualize the response dynamics. To inhibit specific ion channels, Piezo inhibitor GsMTx4 (5 μM; MCE, #HY-P1410) or TRPV inhibitor GSK205 (10 μM; MCE, # HY-120691A) was added 30 min before probe loading; control groups received equivalent dimethyl sulfoxide. Image sequences were analyzed using ImageJ v1.54, with regions of interest defined per cell and Δ*F*/*F*_0_ calculated as (F_t − *F*_0_)/*F*_0_, where *F*_0_ represented baseline fluorescence and F_t represented fluorescence at any time point. To ensure the validity of the Δ*F*/*F*_0_ comparisons across various conditions and mouse strains, we verified that the baseline fluorescence (*F*_0_) remained consistent, with no statistically significant differences observed between genotypes or treatment group.

### Flow cytometry

CD8^+^ T cells from in vitro culture or mouse tumor tissues were prepared as single-cell suspensions using a 70-μm cell strainer (Falcon, #352350). Tumor samples were first digested with collagenase IV (1 mg/ml; Sigma-Aldrich, #C5138) and deoxyribonuclease I (0.1 mg/ml; Sigma-Aldrich, #D5025) at 37°C with shaking for 30 min, followed by red blood cell lysis. All samples were washed with PBS and counted, and 1 × 10^6^ cells were plated in 96-well plates. LIVE/DEAD Fixable Aqua dye (Invitrogen, #L34957) was applied at 23° to 25°C in the dark for 20 min to exclude dead cells, followed by surface staining with fluorochrome-conjugated antibodies: CD3ε (BioLegend, #100312, 1:200), CD8α (#100732, 1:200), PD-1 (#135210, 1:200), TIM-3 (#134007, 1:200), CD69 (#104508, 1:200), and CD28 (#102112, 1:200) at 4°C avoiding light exposure for 30 min. After washing twice with PBS, cells were fixed and permeabilized (30-min fixation, 15-min permeabilization) and then stained intracellularly with anti-TNFα (BioLegend, #506304, 1:200) and anti–IFN-γ (#505806, 1:200) antibodies at 4°C avoiding light for 45 min. Cells were resuspended in PBS (2% FBS), and data were acquired on a BD LSRFortessa flow cytometer with at least 100,000 events per sample. Instrument voltage and compensation were calibrated daily using BD FACSDiva CS&T beads. Data were analyzed using FlowJo v10.9 software with gating strategy: singlet → live → CD3^+^CD8^+^ → target subsets (PD-1^+^TIM-3^+^ or TNFα^+^IFN-γ^+^). Background was determined using isotype controls.

### Cytotoxicity assays

To assess CD8^+^ T cell cytotoxicity, 3LL-OVA cells expressing OVA antigen were used as target cells. Before each coculture experiment, the stable expression of the OVA antigen and its presentation on H-2K^b^ were verified in 3LL-OVA cells via flow cytometry to ensure a consistent target population (>90% positivity). Target cells were plated in 96-well U-bottom plates at 1 × 10^4^ cells per well 24 hours earlier to ensure consistent adhesion and viability. OT-1 CD8^+^ T cells activated with αCD3/αCD28 antibodies for 48 hours were harvested, washed, and counted. Effector and target cells were cocultured at E:T ratios of 10:1, 5:1, and 1:1 in 200-μl total volume per well at 37°C, 5% CO_2_ for 6 hours. Control wells included target spontaneous lysis (targets + medium) and maximum lysis (targets + 1% Triton X-100). Following this, 50 μl of supernatant was transferred to a new plate, and lactate dehydrogenase (LDH) release was determined using the LDH Cytotoxicity Assay Kit (Promega, #G1780) per instructions at 490-nm absorbance [optical density at 490 nm (OD_490_)]. Cytotoxicity (%) was calculated as [(OD_exp − OD_spontaneous) / (OD_max − OD_spontaneous)] × 100%.

### Measurement of acetyl-CoA levels

Nuclear and cytoplasmic fractions of CD8^+^ T cells were isolated using a nuclear/cytosol fractionation kit (BioVision, #K266). Acetyl-CoA concentrations were quantified using an acetyl-CoA fluorometric assay kit (Sigma-Aldrich, #MAK039) per the manufacturer’s instructions. Fluorescence was measured at Ex/Em = 535/587 nm on a microplate reader, and concentrations were normalized to total protein content as determined by BCA assay.

### WB analysis and co-IP

Cells were lysed on ice in radioimmunoprecipitation assay buffer (Thermo Fisher Scientific, #89901), and lysates were centrifuged at 12,000*g* for 15 min. The protein (supernatant) concentration was determined using the BCA Kit (Thermo Fisher Scientific, #23227). Subsequently, 30 μg of protein per sample were denatured in 4× SDS loading buffer (with 5% β-mercaptoethanol) at 95°C for 5 min and separated by 10% SDS-PAGE and then transferred to polyvinylidene difluoride membranes (Millipore, #IPVH00010) at 100 V for 90 min (wet transfer, Bio-Rad Mini Trans-Blot). After blocking, membranes were blocked in 5% nonfat milk in TBST at 23° to 25°C for 1 hour and then incubated with primary antibodies (1:1000) against LOXL4, Piezo1, FAK, pFAK^Y397^, YAP1, pYAP1, YBX1, KAT2A, ACLY, and β-actin at 4°C overnight, followed by probing with HRP-conjugated goat anti-rabbit or goat anti-mouse IgG (1:5000; Cell Signaling Technology) at 23° to 25°C for 1 hour, and signals were visualized using ECL reagent (Thermo Fisher Scientific, #32106) and analyzed using Image J.

Co-IP was conducted in human embryonic kidney (HEK) 293T cells (ATCC, #CRL-3216). Cells were transfected with HA-YBX1 and Flag-CREBBP, Flag-ACLY, Flag-KAT2A, Flag-KAT2B, Flag-KAT5, or Flag-KAT6A using Lipofectamine 3000 (Invitrogen, #L3000001). After 36 hours, cells were lysed, and 500 μg of total protein was incubated with 25 μl of Flag M2 magnetic beads (Sigma-Aldrich, #F1804) at 4°C with rotation for 2 hours. Immune complexes were washed with lysis buffer (1 ml each), eluted in 40 μl 2× SDS sample buffer at 95°C for 5 min, and analyzed by Western blot (WB) for HA-YBX1 and Flag-ACLY/KAT2A interactions; 10% input served as control.

### RNA extraction and quantification

Cells or tissue samples were rinsed with PBS and immediately lysed in TRIzol reagent (Thermo Fisher Scientific, #15596018) to isolate total protein. RNA concentration and purity were measured using NanoDrop 2000 (A260/A280 ratio 1.8-2.0) and integrity confirmed by 1% agarose gel electrophoresis. RNA (1 μg) was reverse transcribed using HiScript III RT SuperMix for qPCR (Vazyme, #R323-01). Real-time qPCR was conducted using ChamQ Universal SYBR qPCR Master Mix (Vazyme, #Q111-02) on an ABI 7500 Fast Real-Time PCR System in 20-μl reactions. Gene expression, relative to glyceraldehyde-3-phosphate dehydrogenase, was calculated using the 2^-ΔΔCt^ method.

### Luciferase assays

To assess regulation of target gene promoter activity by YBX1 or YAP1, promoter fragments (∼2 kb upstream of TSS) from mouse YBX1, PD-1, HAVCR2, CD69, and TCF7 genes were amplified and cloned into pGL4.10[luc2] vector (Promega, #E6651) via KpnI/XhoI digestion. HEK293T cells were plated at 2 × 10^5^ cells per well and cultured for 24 hours to 70 to 80% confluence. Cells were cotransfected with 500 ng of promoter-driven pGL4.10 reporter, 250 ng of YAP1 or YBX1 expression plasmid, and 50 ng of Renilla control (pRL-TK; Promega, #E2241) using Lipofectamine 3000. Control groups received equivalent empty vector (pcDNA3.1). Transfection was performed per Lipofectamine 3000 protocol (P3000: DNA = 2 μl:1 μg) in Opti-MEM. After 36 hours, Luciferase activity was analyzed using the Dual-Luciferase Reporter Assay System (Promega, #E1960).

### CUT&Tag analysis

CUT&Tag was conducted using the EpiCypher CUTANA Hyperactive pG-Tn5 Transposase Kit (EpiCypher, #15-1017) per the manufacturer’s instructions. For each group, 1 × 10^5^ CD8^+^ T cells activated with αCD3/αCD28 for 48 hours were fixed to concanavalin A magnetic beads, sequentially incubated with primary and secondary antibodies. Antibodies used included YAP1 (Cell Signaling Technology, #14074), YBX1 (Abcam, #ab12148), H3K27ac (Cell Signaling Technology, #8173), and H3K4me3 (Cell Signaling Technology, #9751) at 1:100 dilution. Cells were probed with antibodies at 4°C overnight with rotation, followed by pAG-Tn5 transposase complex at 23° to 25°C for 1 hour (37°C, 1000 rpm activation). Reactions were terminated, DNA lysed, purified, and fragments recovered using VAHTS DNA Clean Beads (Vazyme, #N411) NEBNext Ultra II DNA Library Prep Kit for Illumina (NEB, #E7645) was used for library preparation. Results were visualized with deepTools v3.5.2 and IGV v2.17.3, with signals normalized as RPKM.

### ChIP-qPCR

Adhering to the guidelines of the SimpleChIP Enzymatic Chromatin IP Kit (Cell Signaling Technology, #9005), 2 × 10^6^ CD8^+^ T cells activated with αCD3/αCD28 for 48 hours were cross-linked with 1% formaldehyde at 23° to 25°C for 10 min and quenched with glycine for 5 min. Nuclei were lysed, and chromatin was sonicated (Bioruptor Pico, 30 s on/off, 10 cycles) to 200– to 600–base pair (bp) fragments. Target antibodies (YAP1, YBX1, or H3K27ac; 5 μg per sample) and Protein G magnetic beads were loaded and incubated overnight at 4°C with rotation. Immune complexes were washed with low- and high-salt buffers, reverse cross-linked at 65°C for 2 hours, and DNA was purified. Recovered DNA was used as template for qPCR (ABI 7500, SYBR Green Mix; Vazyme, #Q111-02). Primers targeted enriched promoter regions of *Cd69*, *Cd28*, *Tcf7*, *Gzmb*, *Prf1*, *Pd-1*, *Tim-3*, *Ctla4*, and *Tox*. Enrichment was expressed as % Input. Each experiment was independently repeated three times.

### ATAC-seq

ATAC-seq was conducted following the previous literature (*72*). For each group, 5 × 10^4^ CD8^+^ T cells treated with different ECM stiffness for 48 hours were rinsed and resuspended in 50 μl of transposition reaction mix and incubated at 37°C for 30 min. Products were purified with DNA Clean Beads and libraries amplified (12 to 14 cycles). Library quality was assessed on Agilent 2100 Bioanalyzer (peak 200 to 800 bp) and sequenced on Illumina NovaSeq 6000 (PE150).

### RNA-seq analysis

DEGs between CD8^+^ T cells seeded on soft (0 μg/ml mLX4) versus stiff (5 μg/ml mLX4) gels, as well as between *Piezo1*^dko^ CD8^+^ T cells overexpressing YBX1 or not were determined using RNA-seq analysis. In brief, total RNA was isolated using TRIzol (Thermo Fisher Scientific, #15596018), and samples with RIN ≥ 7.0 were collected for library preparation (Illumina, #RS-122-2101). Libraries were sequenced on Illumina NovaSeq 6000 (PE150, ∼50 M reads per sample). Raw data were trimmed with Trimmomatic v0.39, aligned to mm10 (Ensembl release 109) using STAR v2.7.10a, and gene expression quantified with FeatureCounts v2.0.3. Differential expression was determined using DESeq2 v1.40.2 (|log_2_FC| > 1, FDR < 0.05).

### Statistical analysis

All experiments were independently repeated a minimal of six times, with results expressed as means ± SD. Data were analyzed using Prism v10.0 (GraphPad Software, USA), R v4.4.0 (R Foundation), and FlowJo v10.9 (BD Biosciences). Two-group comparisons were performed using two-tailed Student’s *t* test; multiple groups were compared using one-way or two-way analysis of variance (ANOVA), followed by Tukey’s (for one-way ANOVA) or Sidak’s (for two-way) post hoc tests. Survival curves were generated using the Kaplan-Meier method and compared with log-rank tests. Statistical significance was set at *P* < 0.05. All raw data, statistical source files, and custom R scripts are available upon reasonable request to ensure reproducibility and transparency.
