# Supplementary material for: Targeting LOXL4-driven matrix stiffening with acetyldigoxin restores CD8+ T cell function in lung cancer
Source: Sci Adv. 2026 Sep 11;12(37):eaef1694. doi: 10.1126/sciadv.aef1694 (PMC13564810; doi:10.1126/sciadv.aef1694)
Supplement: Supplementary file 1 [file sciadv.aef1694_sm.pdf]

Science Advances
AAAS

Supplementary Materials for

Targeting LOXL4-driven matrix stiffening with acetyldigoxin restores $CD8^+$ T cell function in lung cancer

Xuyu Gu *et al.*

Corresponding author: William C. Cho, williamcscho@gmail.com, chocs@ha.org.hk;
Qiyu Fang, qyfang@tongji.edu.cn

Sci. Adv. **12**, eaef1694 (2026)
DOI: 10.1126/sciadv.aef1694

The PDF file includes:

Figs. S1 to S14
Supplementary file S1
Legend for supplementary file S2

Other Supplementary Material for this manuscript includes the following:

Supplementary file S2

Supplementary File 1. Supplementary Figures

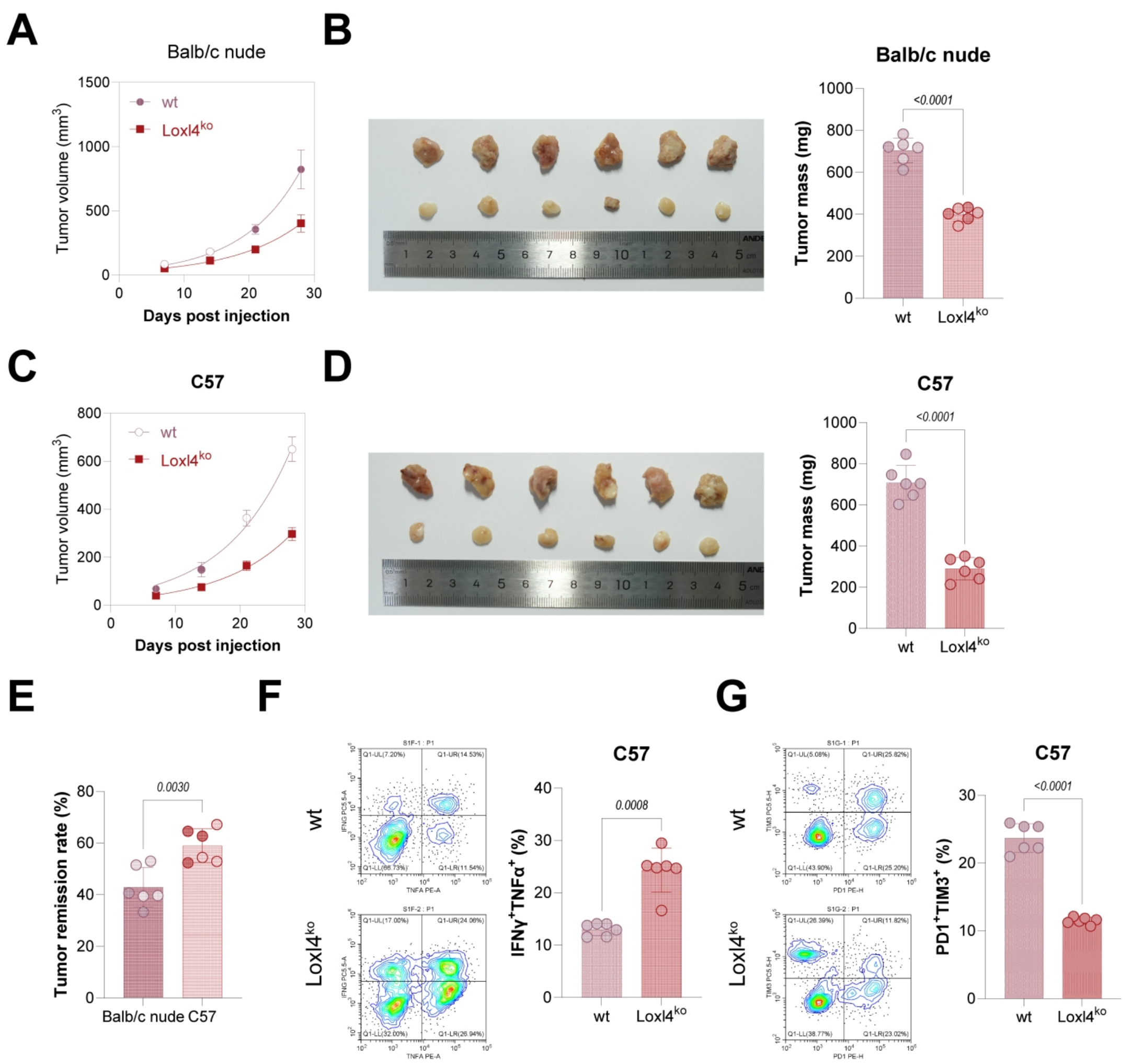

Fig. S1. *Loxl4* knockout reduces tumorigenic activity of 3LL cells *in vivo*. WT or *Loxl4*^{ko} mouse 3LL lung cancer cells were implanted into either immunocompromised BALB/c nude mice or immunocompetent C57 mice subcutaneously. A, Weekly volume change in subcutaneous tumors in BALB/c mice; B, Representative images and weight of subcutaneous tumors in BALB/c mice on day 28. C, Weekly volume change in subcutaneous tumors in C57 mice; D, Representative images and weight of subcutaneous tumors in C57 mice on day 28; E, Comparison of tumor growth restriction rate for *Loxl4*^{ko} 3LL cells in C57 mice versus BALB/c nude mice; F-G, Populations of effector CD8^{+} T cells (TNFα^{+}IFNγ^{+}) and exhausted T cells (PD-1^{+}TIM-3^{+}) in tumors formed by WT or *Loxl4*^{ko} 3LL cells in C57 mice. Each group contained six mice. Data are presented as dot plots and bars. Statistical significance was determined using unpaired *t*-tests (B, D-G) or two-way ANOVA followed by Sidak's post-hoc test (A and C). Statistical significance was set at $p < 0.05$.

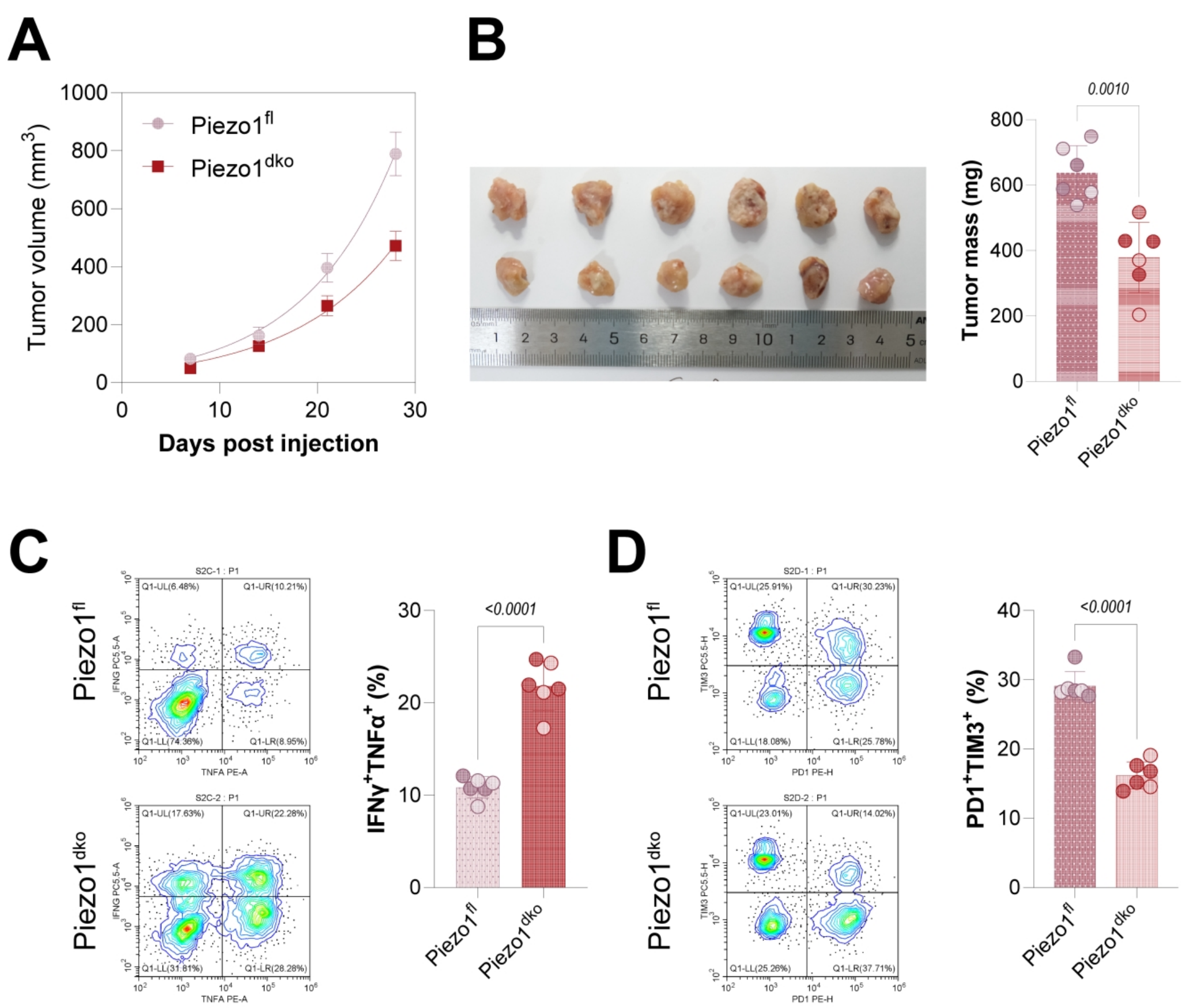

Fig. S2. CD8+ T cell-specific *Piezo1* knockout restricts tumorigenic activity of 3LL cells in mice. *Piezo1*dko and *Cd8a*-Cre mice crossed to generate a CD8+ T cell-specific Piezo1 knockout (*Piezo1*dko) strain, followed by subcutaneous injection of 3LL cells into either *Piezo1*dko or *Piezo1*fl/fl mice. A, Weekly volume change in subcutaneous tumors in *Piezo1*dko or *Piezo1*fl/fl mice; B, Representative images and weight of subcutaneous tumors in *Piezo1*dko or *Piezo1*fl/fl mice on day 28; C-D, Populations of effector CD8+ T cells (TNFα+IFNγ+) and exhausted T cells (PD-1+TIM-3+) in tumors in *Piezo1*dko or *Piezo1*fl/fl mice determined using flow cytometry. Each group contained six mice. Data are presented as dot plots and bars. Statistical significance was determined using unpaired *t*-tests (B-D) or two-way ANOVA followed by Sidak's post-hoc test (A). Statistical significance was set at $p < 0.05$.

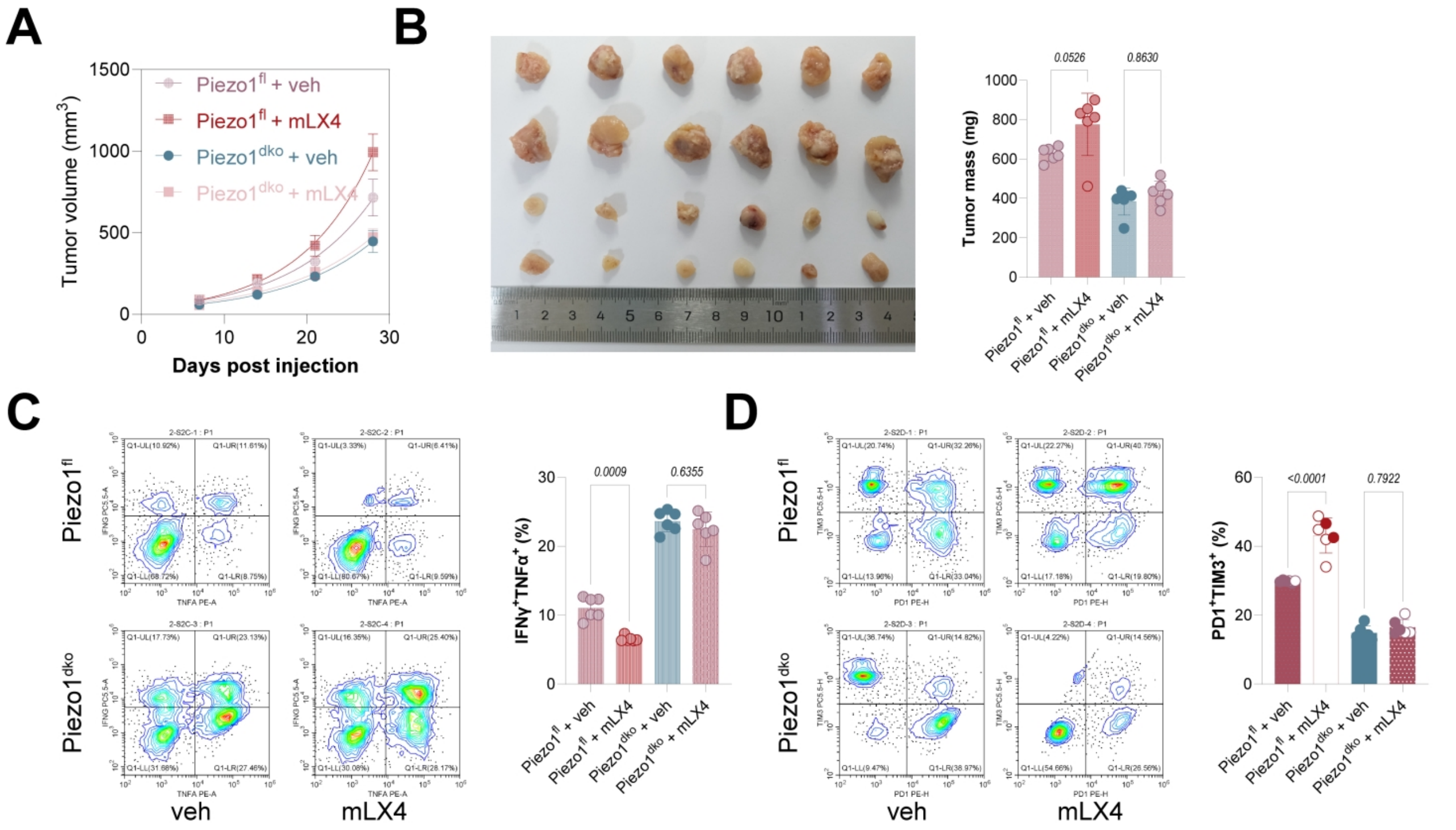

Fig. S3. mLX4 fails to enhance tumorigenic activity of 3LL cells in *Piezo1*^{dko} mice. 3LL cells were implanted into either *Piezo1*^{dko} or *Piezo1*$^{fl/fl}$ mice, followed by intratumoral injection of mLX4 or a control vehicle. A, Weekly volume change in subcutaneous tumors in mice across groups; B, Representative images and weight of subcutaneous tumors in mice on day 28; C-D, Populations of effector CD8$^+$ T cells (TNFα$^+$IFNγ$^+$) and exhausted T cells (PD-1$^+$TIM-3$^+$) in tumors in mice across groups determined using flow cytometry. Each group contained six mice. Data are presented as dot plots and bars. Statistical significance was determined using one-way ANOVA followed by Tukey's post-hoc test (B-D) or two-way ANOVA followed by Sidak's post-hoc test (A). Statistical significance was set at $p < 0.05$.

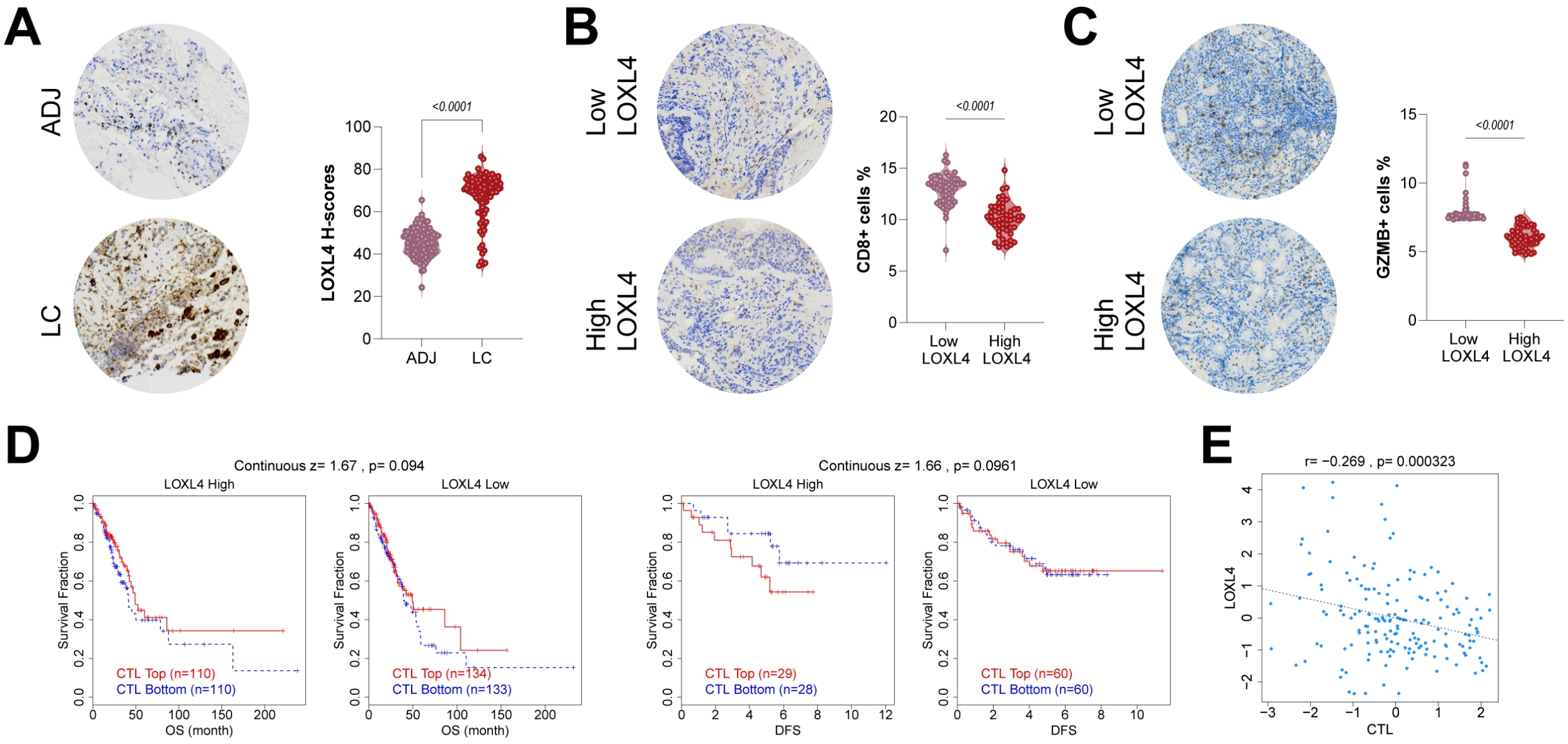

Fig. S4. LOXL4 is negatively correlated with CTLs in lung cancer patients. A, IHC analysis of LOXL4 staining intensity in tumor tissues and adjacent non-tumor tissues from 104 lung cancer patients. B-C, The 104 patients were stratified into high- and low-expression groups based on the median LOXL4 expression level. The numbers of $CD8^+$ and $GZMB^+$ cells were assessed by IHC. D-E, Correlation analysis between *Loxl4* expression levels and the infiltration of $CD8^+$ T cells and CTLs in the TCGA-LUAD and TCGA-LUSC datasets, determined using the CIBERSORT algorithm. Statistical significance was determined using Student's t tests (A-C). Statistical significance was set at $p < 0.05$.

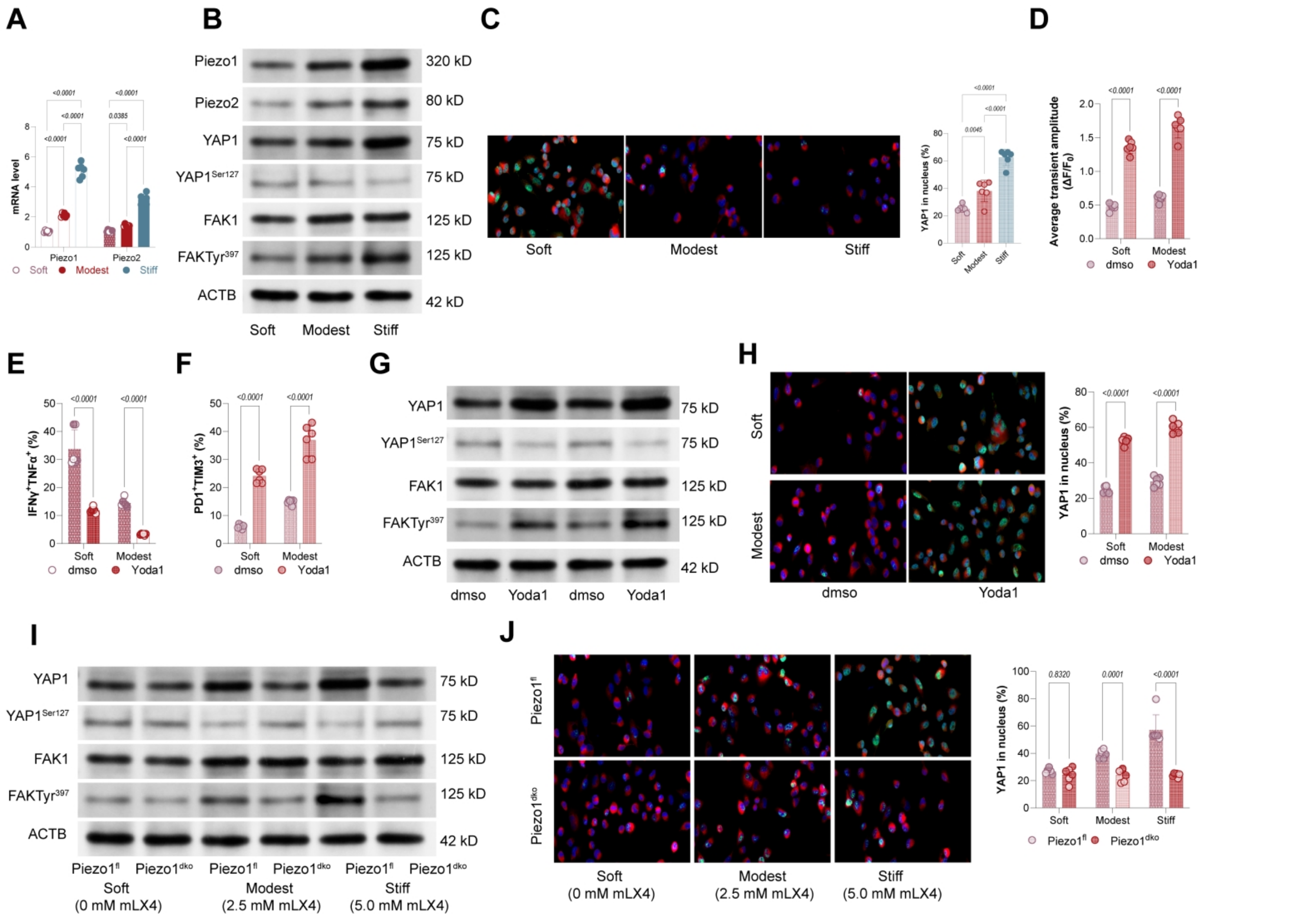

Fig. S5. FAK1-YAP1 activation in response to Piezo1-mediated mechanical force. A, mRNA expression of *Piezo1* and *Piezo2* in CD8$^+$ T cells cultured in soft, moderate, or stiff gels (0, 2.5, or 5.0 µg/mL mLX4) determined using qPCR analysis; B, protein levels of Piezo1, Piezo2, YAP1, FAK1, and phosphorylation of YAP1^{Ser127} and FAK^{Tyr397} in CD8$^+$ T cells cultured in soft, moderate, or stiff gels (0, 2.5, or 5.0 µg/mL mLX4) determined using WB analysis; C, YAP1 nuclear localization in CD8$^+$ T cells cultured in soft, moderate, or stiff gels (0, 2.5, or 5.0 µg/mL mLX4) determined using immunofluorescence staining. Yoda1, a potent Piezo1 agonist, was added to the cultures of CD8$^+$ T cells seeded on soft or moderate gels (0 or 2.5 µg/mL mLX4). D, Quantification of average Ca^{2+} transient amplitude ($\Delta F/F_0$) in CD8$^+$ T cells after the addition of Yoda1; E-F, Populations of effector CD8$^+$ T cells (TNFα$^+$IFNγ$^+$) and exhausted T cells (PD-1$^+$TIM-3$^+$) after the addition of Yoda1 determined using flow cytometry; G, Protein levels and phosphorylation of FAK1 and YAP1 in CD8$^+$ T cells after the addition of Yoda1; H, YAP1 nuclear localization in CD8$^+$ T cells after the addition of Yoda1 determined using immunofluorescence staining; I, Protein levels and phosphorylation of FAK1 and YAP1 in *Piezo1*^{dko} or *Piezo1*$^{fl/fl}$ CD8$^+$ T cells cultured in soft,

moderate, or stiff gels (0, 2.5, or 5.0 μg/mL mLX4) determined using WB analysis; J, YAP1 nuclear localization in *Piezo1*^{dko} or *Piezo1*$^{fl/fl}$ $CD8^+$ T cells cultured in soft, moderate, or stiff gels (0, 2.5, or 5.0 μg/mL mLX4) determined using immunofluorescence staining. Cell experiments repeated six times. Data are presented as dot plots and bars. Statistical significance was determined using one-way ANOVA followed by Tukey's post-hoc test (C) or two-way ANOVA followed by Sidak's post-hoc test (A, D, E, F, H, and J). Statistical significance was set at $p < 0.05$.

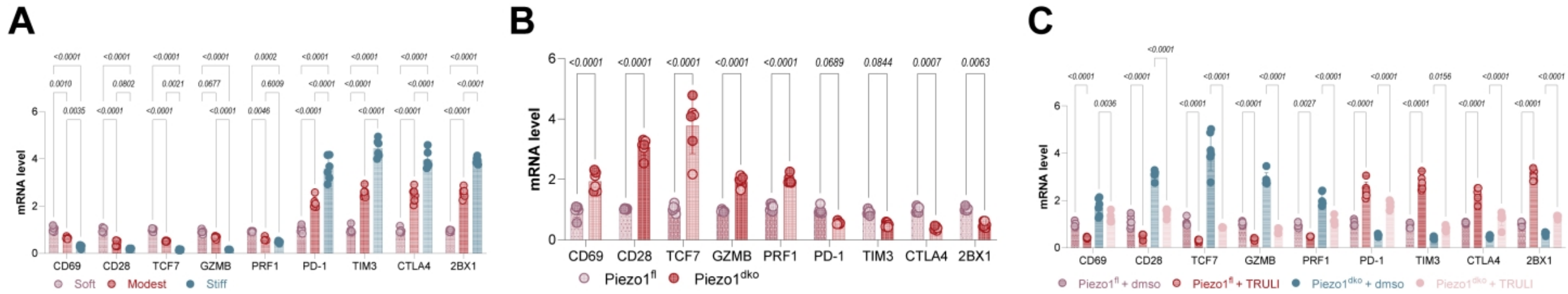

Fig. S6. LOXL4-mediated ECM stiffening promotes Piezo1-dependent expression of CD8$^+$ T cell exhaustion markers. A, mRNA expression of CD8$^+$ T cell effector markers *Cd69, Cd28, Tcf7, Gzmb,* and *Prf1* and exhaustion indicators *Pd-1 (Pdcd1), Tim-3 (Gavr2), Ctla4,* and *Tox* in CD8$^+$ T cells cultured in soft, moderate, or stiff gels (0, 2.5, or 5.0 μg/mL mLX4) determined using qPCR analysis; B, mRNA expression of these T cell effector or exhaustion markers in CD8$^+$ T cells from *Piezo1*^{dko} or *Piezo1*$^{fl/fl}$ mice determined using qPCR analysis; C, mRNA expression of these T cell effector or exhaustion markers in CD8$^+$ T cells from TRULI-treated *Piezo1*^{dko} or *Piezo1*$^{fl/fl}$ mice determined using qPCR analysis. Cell experiments repeated six times. Data are presented as dot plots and bars. Statistical significance was determined using two-way ANOVA followed by Sidak's post-hoc test (A-C). Statistical significance was set at $p < 0.05$.

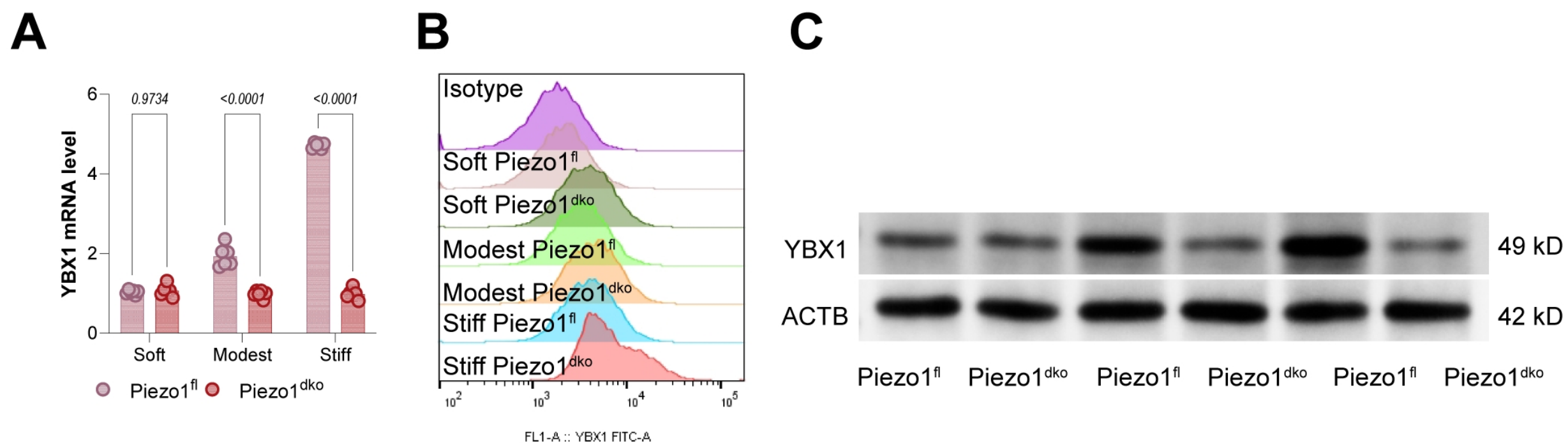

Fig. S7. mLX4-mediated mechanical force fails to modulate YBX1 expression in *Piezo1*^{dko} T cells. CD8^{+} T cells from *Piezo1*^{dko} or *Piezo1*$^{fl/fl}$ mice were cultured in soft, moderate, or stiff gels (0, 2.5, or 5.0 μg/mL mLX4), followed by qPCR (A), flow cytometry (B), and WB analysis (C) to detect YBX1 levels. Cell experiments repeated six times. Data are presented as dot plots and bars. Statistical significance was determined using two-way ANOVA followed by Sidak's post-hoc test (A). Statistical significance was set at $p < 0.05$.

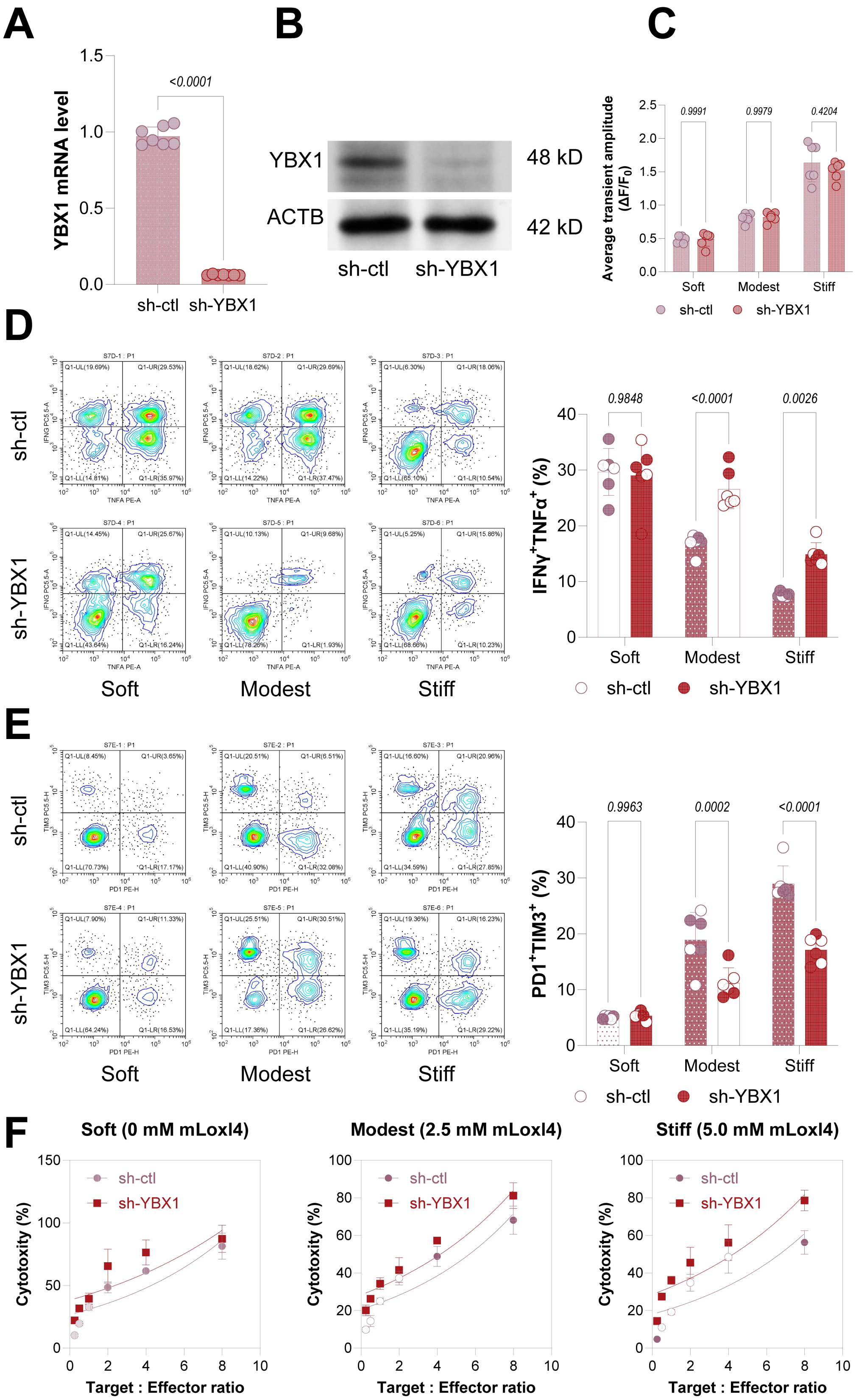

Fig. S8. YBX1 deficiency attenuates mechanical force-mediated CD8⁺ T cell exhaustion. A-B, mRNA and protein levels of YBX1 in CD8⁺ T cells after transduction of the YBX1 shRNA determined using qPCR and WB analyses; C, Quantification of average Ca^{2+} transient amplitude

($\Delta F/F_0$) in $CD8^+$ T cells following YBX1 knockdown; D-E, Populations of effector $CD8^+$ T cells ($TNF\alpha^+IFN\gamma^+$) and exhausted T cells ($PD\text{-}1^+TIM\text{-}3^+$), with or without YBX1 silencing, after culture on soft, moderate, and stiff gels determined using flow cytometry; F, Cytotoxicity assays of T cells with YBX1 loss against 3LL cells. Cell experiments repeated six times. Data are presented as dot plots and bars. Statistical significance was determined using unpaired *t*-tests (A) or two-way ANOVA followed by Sidak's post-hoc test (C-F). Statistical significance was set at $p < 0.05$.

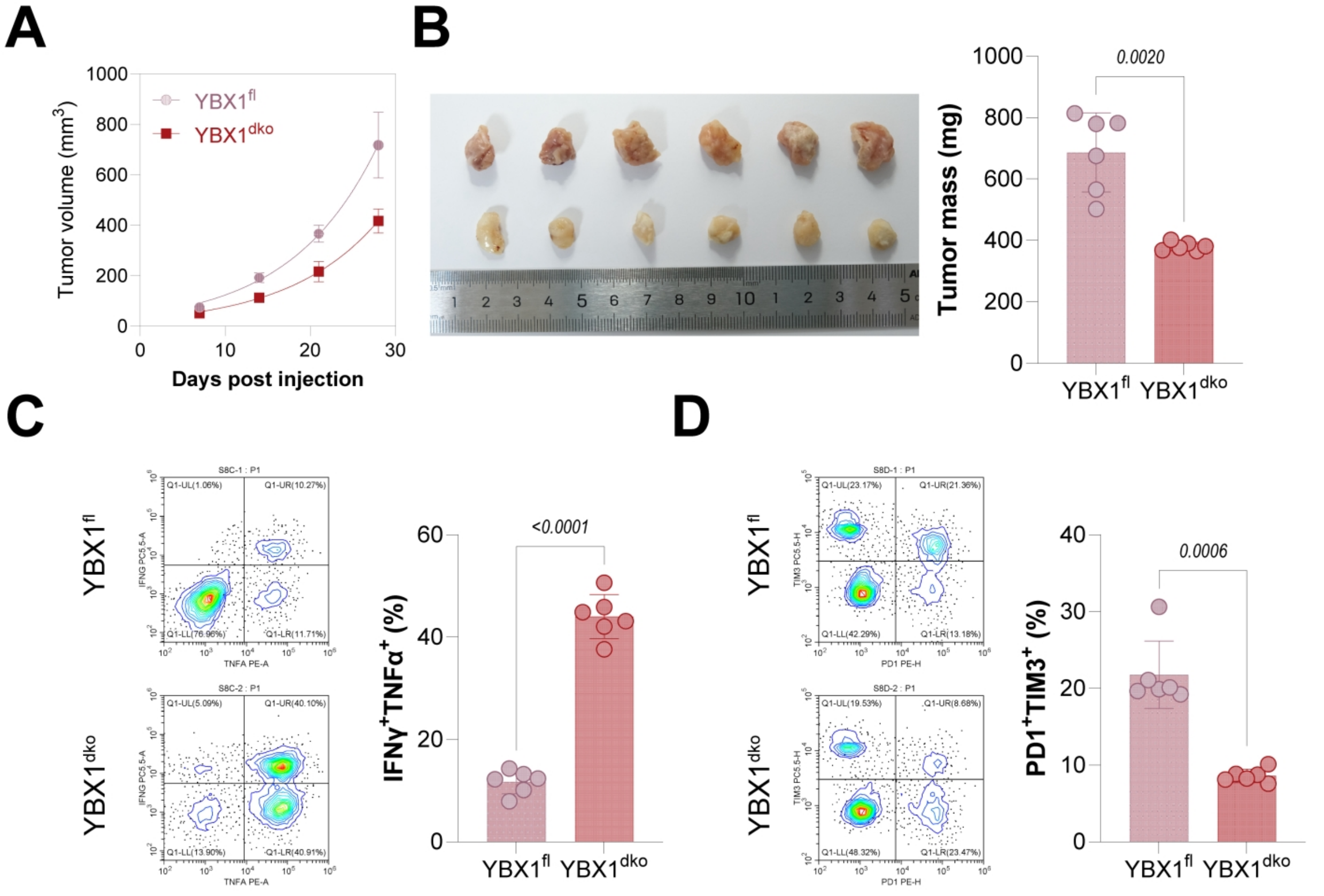

Fig. S9. CD8$^+$ T cell-specific *Ybx1* knockout restricts tumorigenic activity of 3LL cells in mice. *Ybx1*$^{fl/fl}$ and *Cd8a*-Cre mice were allowed to mate to generate a CD8$^+$ T cell-specific *Ybx1* knockout (*Ybx1*^{dko}) strain, followed by subcutaneous injection of 3LL cells into either *Ybx1*^{dko} or *Ybx1*$^{fl/fl}$ mice. A, Weekly volume change in subcutaneous tumors in *Ybx1*^{dko} or *Ybx1*$^{fl/fl}$ mice; B, Representative images and weight of subcutaneous tumors in *Ybx1*^{dko} or *Ybx1*$^{fl/fl}$ mice on day 28; C-D, Populations of effector CD8$^+$ T cells (TNFα$^+$IFNγ$^+$) and exhausted T cells (PD-1$^+$TIM-3$^+$) in tumors in *Ybx1*^{dko} or *Ybx1*$^{fl/fl}$ mice determined using flow cytometry. Each group contained six mice. Data are presented as dot plots and bars. Statistical significance was determined using unpaired *t*-tests (B-D) or two-way ANOVA followed by Sidak's post-hoc test (A). Statistical significance was set at $p < 0.05$.

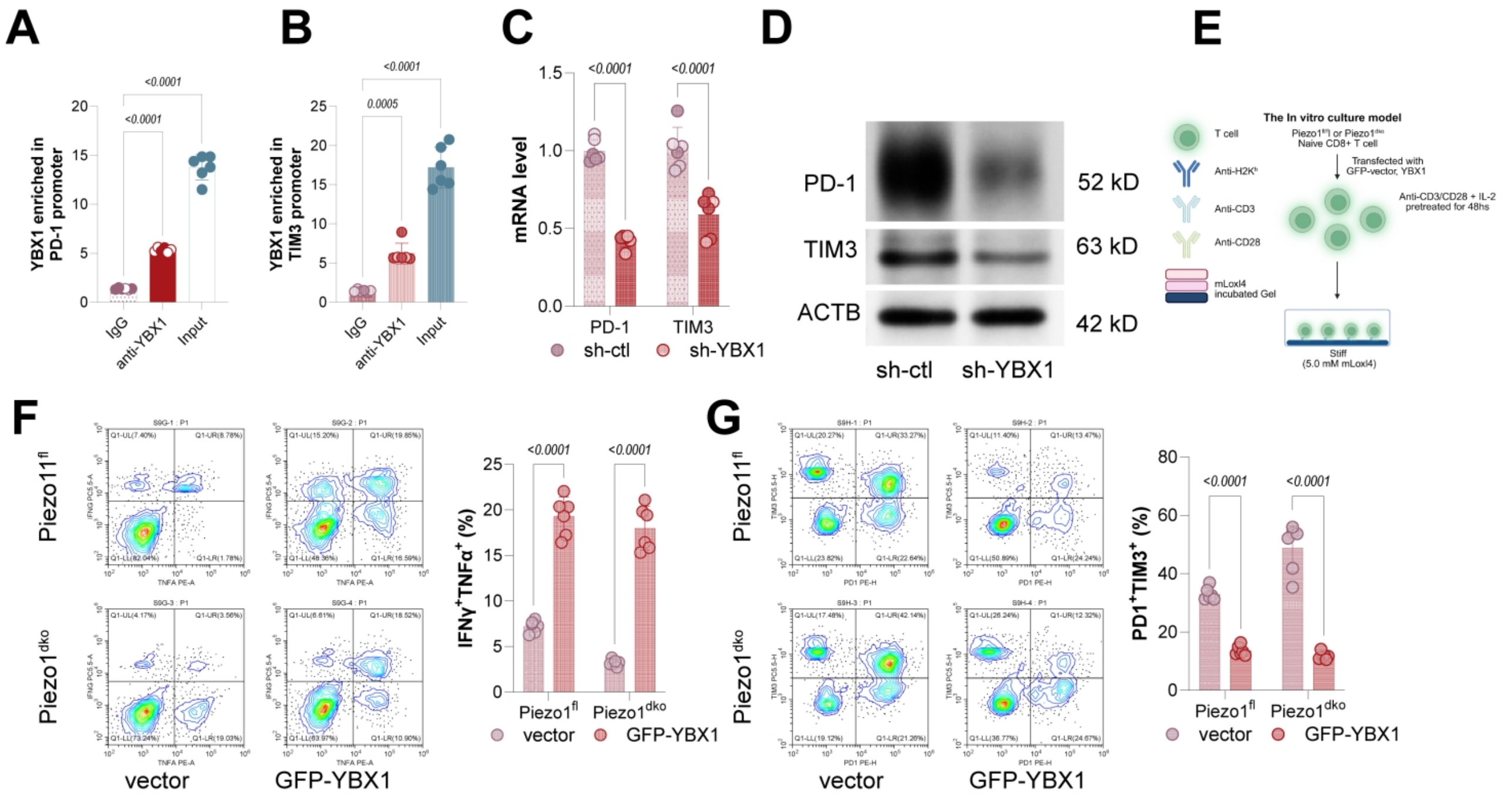

Fig. S10. YBX1 interacts with *Pdcd1* and *Havcr2* promoters and upregulates their expression. A-B, ChIP with anti-YBX1 followed by qPCR to examine the binding of YBX1 to PD-1 and TIM-3 promoter segments; C-D, mRNA and protein levels of PD-1 and TIM-3 in YBX1-knockdown $CD8^+$ T cells determined using qPCR and WB analyses; E, YBX1 overexpression was induced in *Piezo1*^{dko} or *Piezo1*$^{fl/fl}$ $CD8^+$ T cells, which were then cultured in stiff gels (5 μg/mL mLX4); F-G, Populations of effector $CD8^+$ T cells ($TNF\alpha^+IFN\gamma^+$) and exhausted T cells ($PD\text{-}1^+TIM\text{-}3^+$) in *Piezo1*^{dko} or *Piezo1*$^{fl/fl}$ $CD8^+$ T cells after YBX1 overexpression determined using flow cytometry. Cell experiments repeated six times. Data are presented as dot plots and bars. Statistical significance was determined using one-way ANOVA followed by Tukey's post-hoc test (A and B), or two-way ANOVA followed by Sidak's post-hoc test (C, F, and G). Statistical significance was set at $p < 0.05$.

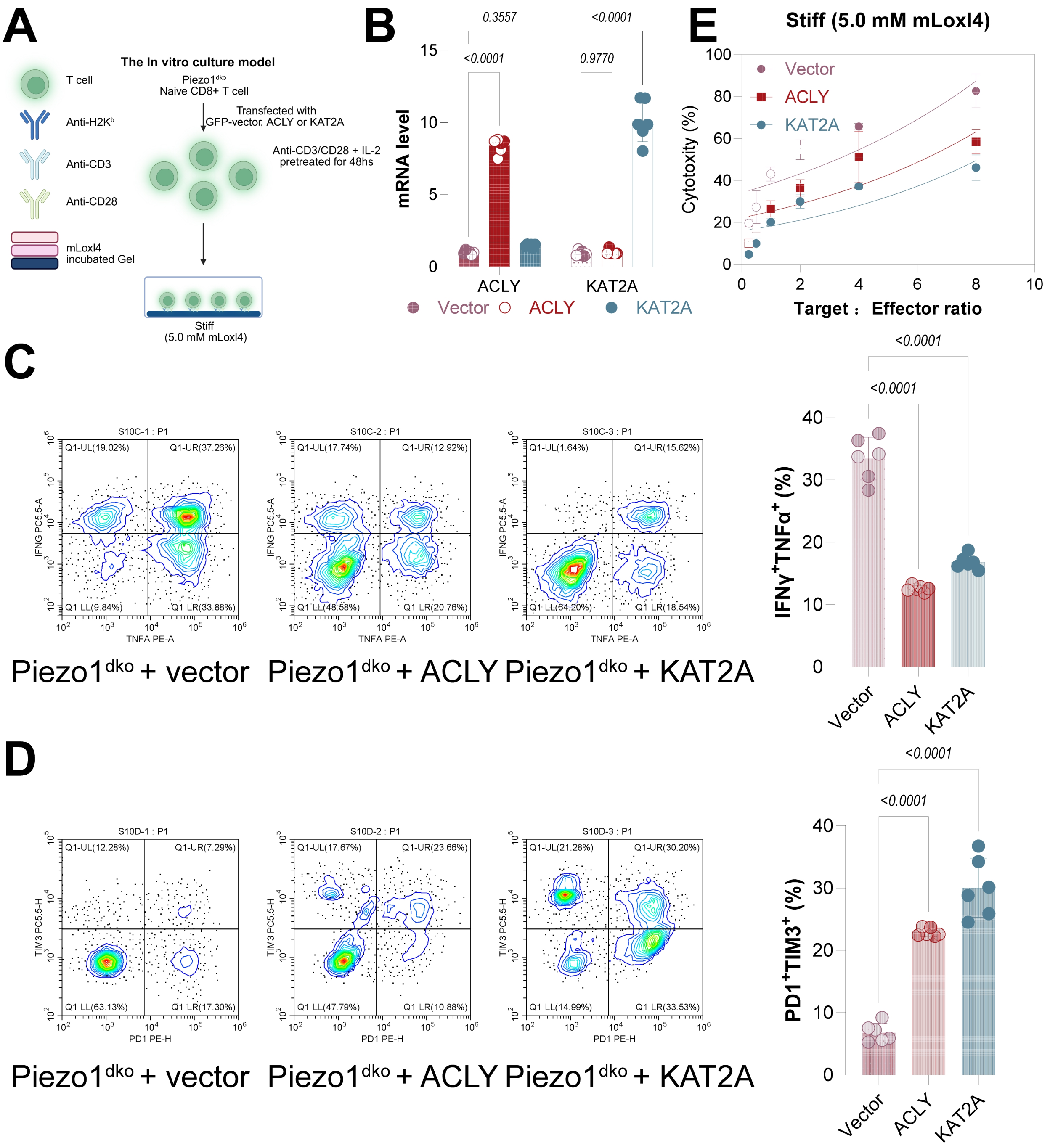

Fig. S11. Overexpression of KAT2A or ACLY promotes CD8⁺ T cell exhaustion. A, *Piezo1*^{dko} CD8⁺ T cells were transfected with ACLY or KAT2A overexpression vectors, activated by αCD3/αCD28 antibodies, and seeded on stiff gels (5.0 µg/mL mLX4); B, qPCR analysis of *Kat2a* and *Acly* mRNA expression in cells after transfection of ACLY or KAT2A overexpression vectors; C-D, Populations of effector CD8⁺ T cells (TNFα⁺IFNγ⁺) and exhausted T cells (PD-1⁺TIM-3⁺) in CD8⁺ T cells upon KAT2A or ACLY overexpression determined using flow cytometry. E, Cytotoxicity assay of T cells with KAT2A or ACLY overexpression against 3LL cells. Cell

experiments repeated six times. Data are presented as dot plots and bars. Statistical significance was determined using one-way ANOVA followed by Tukey's post-hoc test (C-D) or two-way ANOVA followed by Sidak’s post-hoc test (A-B). Statistical significance was set at $p < 0.05$.

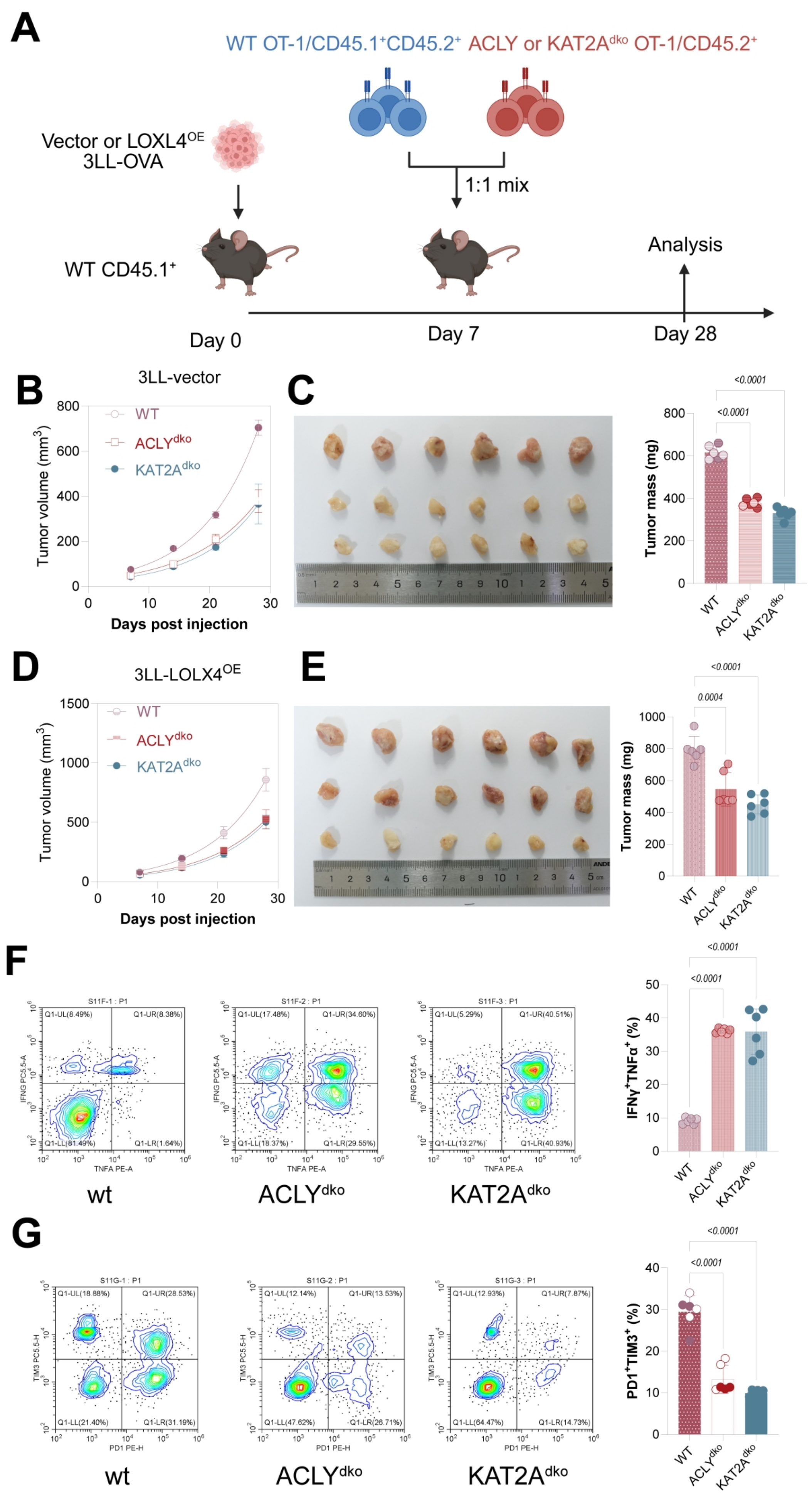

A
WT OT-1/CD45.1⁺CD45.2⁺ ACLY or KAT2Adko OT-1/CD45.2⁺
Vector or LOXL4OE 3LL-OVA
1:1 mix
WT CD45.1⁺
Analysis
Day 0
Day 7
Day 28
B
3LL-vector
WT
ACLYdko
KAT2Adko
Tumor volume (mm3)
Days post injection
C
Tumor mass (mg)
<0.0001
<0.0001
WT
ACLYdko
KAT2Adko
D
3LL-LOLX4OE
WT
ACLYdko
KAT2Adko
Tumor volume (mm3)
Days post injection
E
Tumor mass (mg)
<0.0001
0.0004
F
IFNγ⁺TNFα⁺ (%)
<0.0001
<0.0001
wt
ACLYdko
KAT2Adko
G
PD1⁺TIM3⁺ (%)
<0.0001
<0.0001
wt
ACLYdko
KAT2Adko

Fig. S12. ACLY or KAT2A deficiency enhances anti-tumor immunity. A, 3LL cells (empty vector or LOXL4-overexpressing) were implanted in C57 mice; on day 7, OT1 $CD45.2^{+}$ cells from *Acly*^{dko} or *Kat2a*^{dko} mice were adoptively transferred. Created in BioRender. Gu, X. (2026) https://BioRender.com/g01umjl; B, Weekly volume change in subcutaneous

tumors formed by 3LL cells (empty vector) in mice; C, Representative images and weight of subcutaneous tumors formed by 3LL cells (empty vector) in mice on day 28; D, Weekly volume change in subcutaneous tumors formed by LOXL4-overexpressing 3LL cells in mice; E, Representative images and weight of subcutaneous tumors formed by LOXL4-overexpressing 3LL cells in mice on day 28; F-G, Populations of effector $CD8^{+}$ T cells ($TNF\alpha^{+}IFN\gamma^{+}$) and exhausted T cells ($PD\text{-}1^{+}TIM\text{-}3^{+}$) determined using flow cytometry. Each group contained six mice. Data are presented as dot plots and bars. Statistical significance was determined using one-way ANOVA followed by Tukey's post-hoc test (C, E, F, and G) or two-way ANOVA followed by Sidak’s post-hoc test (B and D). Statistical significance was set at $p < 0.05$.

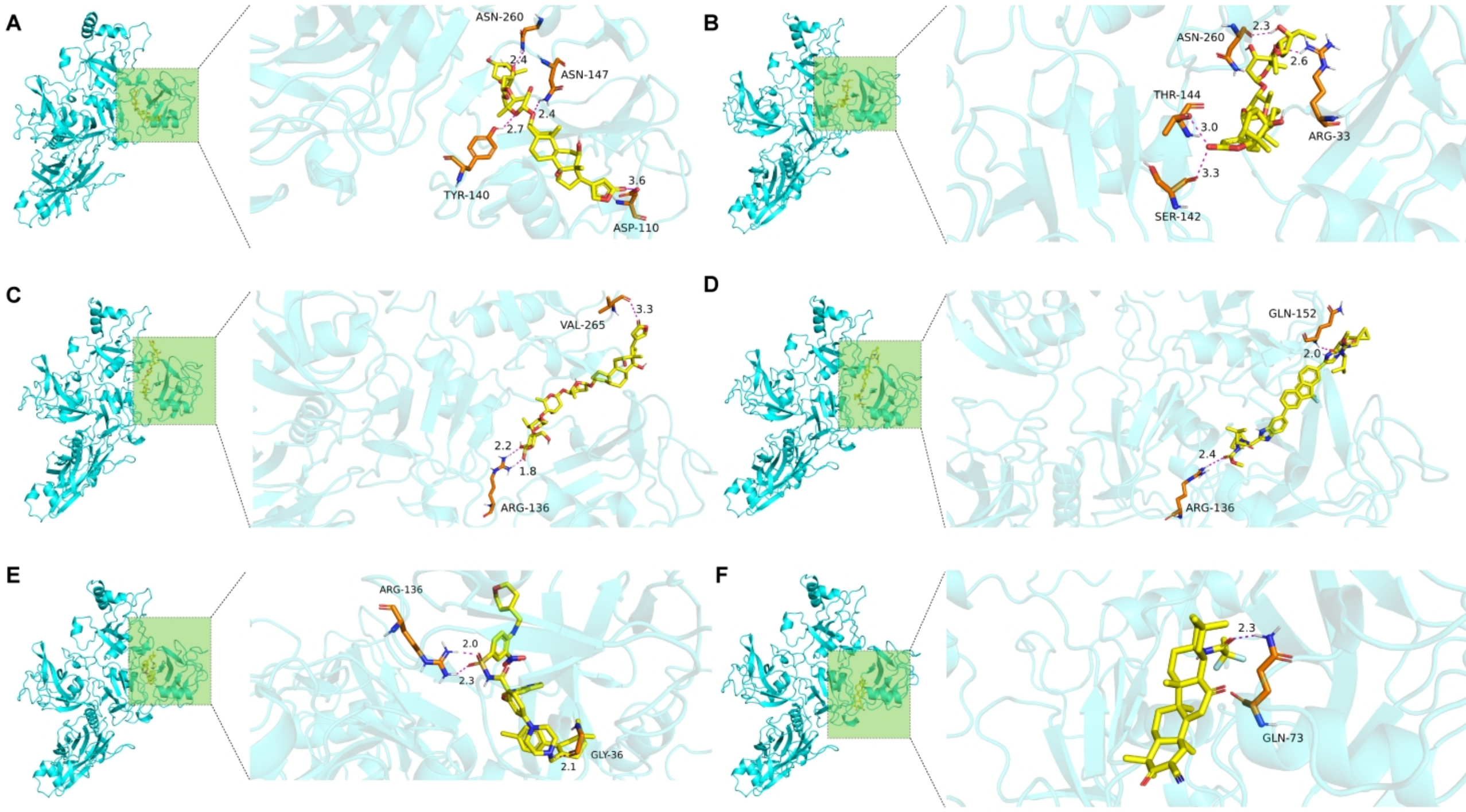

Fig. S13. Molecular docking analysis of FDA-approved compounds potentially binding LOXL4. A-F, Schematic diagram of the interaction between Deslanoside, Ledipasvir, Acetyldigoxin, Omaveloxolone, Venetoclax, and Digoxin and the Scavenger Receptor Cysteine-Rich domain of LOXL4.

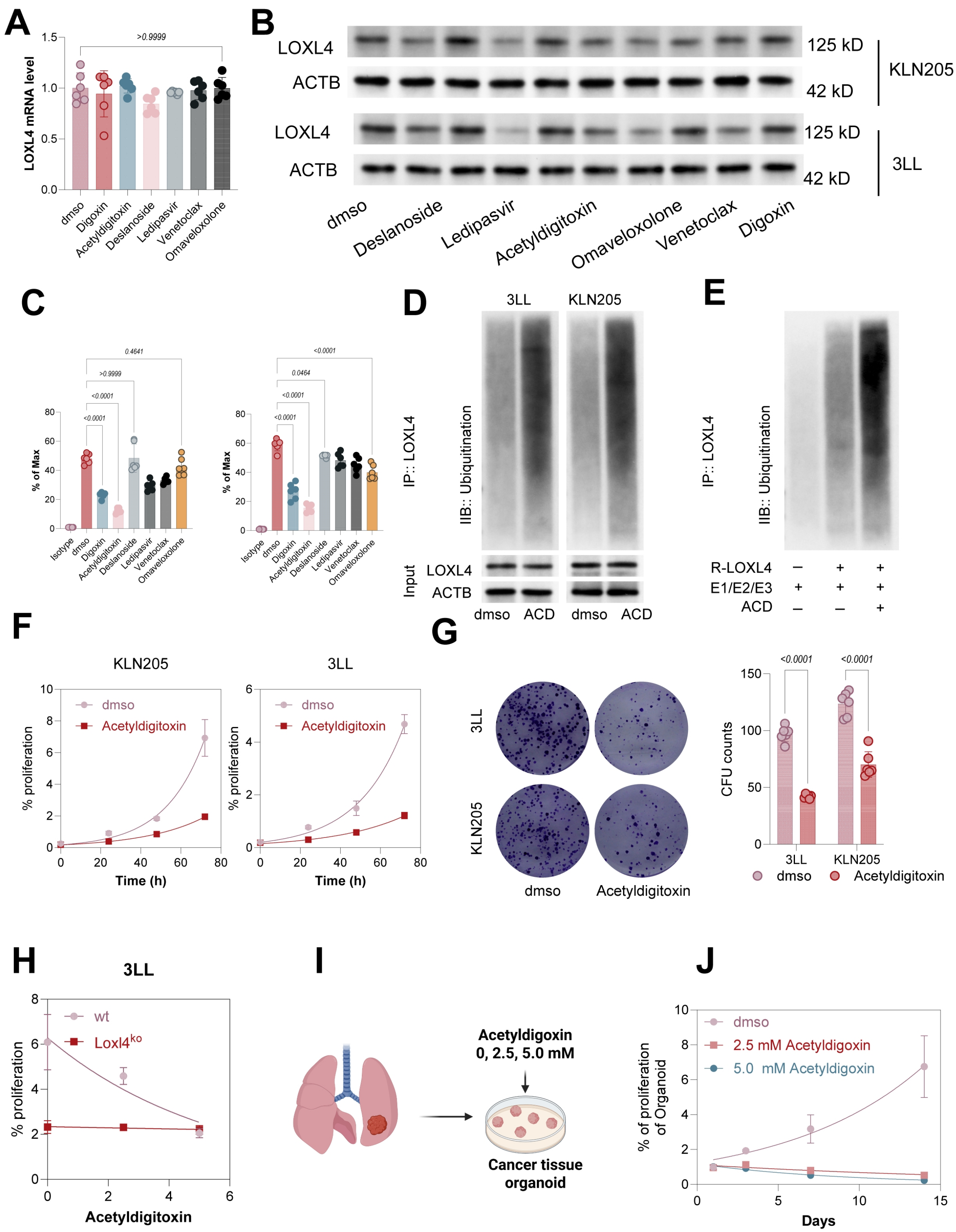

Fig. S14. Acetyldigoxin reduces LOXL4 protein levels and inhibits the growth of mouse lung cancer cells *in vitro*. A-C, qPCR, WB analysis, and flow cytometry analysis of LOXL4 expression in

3LL cells after treatment with DMSO, Deslanoside, Ledipasvir, Acetyldigoxin, Omaveloxolone, Venetoclax, or Digoxin; D, Representative immunoblots of LOXL4 ubiquitination in 3LL and KLN205 cells. Cells were treated with acetyldigoxin (5 mM) for 24 h. LOXL4 was immunoprecipitated (IP) from whole-cell lysates, and poly-ubiquitination levels were detected by WB using an anti-Ubiquitin antibody. E, *In vitro* ubiquitination assay using recombinant LOXL4 protein incubated with E1, E2, and Ubiquitin in the presence or absence of acetyldigoxin. F, Proliferation of 3LL or KLN205 cells after acetyldigoxin treatment determined using CCK-8 assays; G, Colony formation ability of 3LL or KLN205 cells after acetyldigoxin treatment. H, Comparative proliferation analysis of WT versus *Loxl4*^{ko} 3LL cells treated with acetyldigoxin to determine target specificity. I, Establishment of LUAD organoids and treatment with acetyldigoxin for 0, 3, 7, and 14 days. Created in BioRender. Gu, X. (2026) https://BioRender.com/o3rvwpp. J, Assessment of organoid growth activity using the CellTiter-Glo assay. Cell experiments repeated six times. Data are presented as dot plots and bars. Statistical significance was determined using one-way ANOVA followed by Tukey's post-hoc test (A and C) or two-way ANOVA followed by Sidak’s post-hoc test (D, E, F, and H). Statistical significance was set at $p < 0.05$.

Supplementary File 2. Original Blots

5H

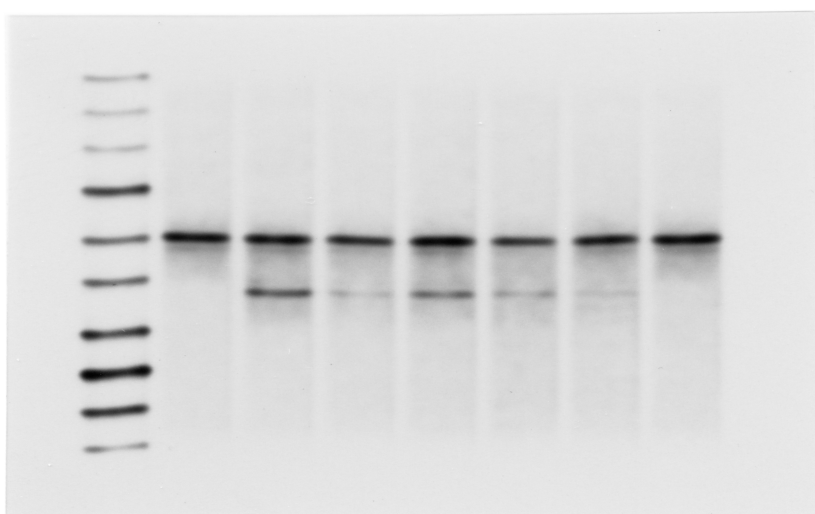

1

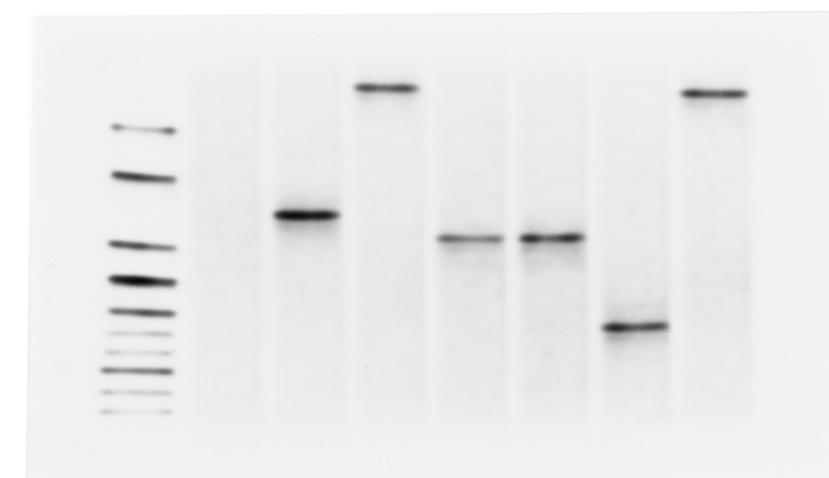

2

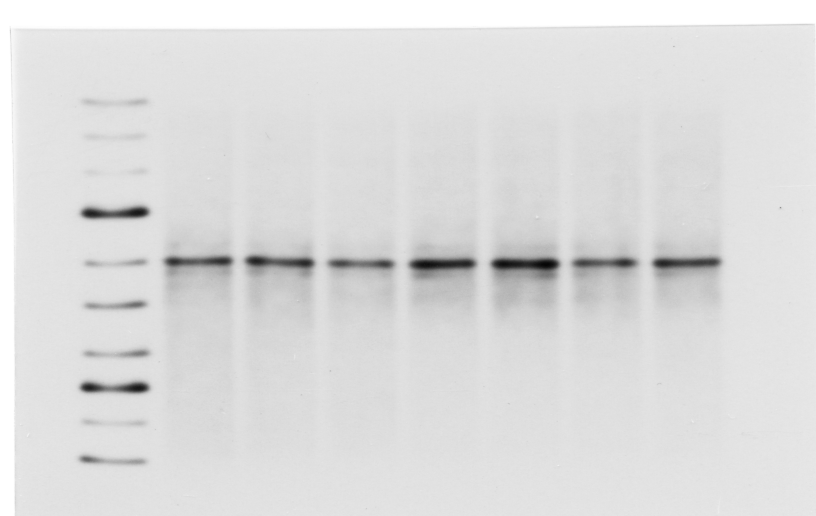

3

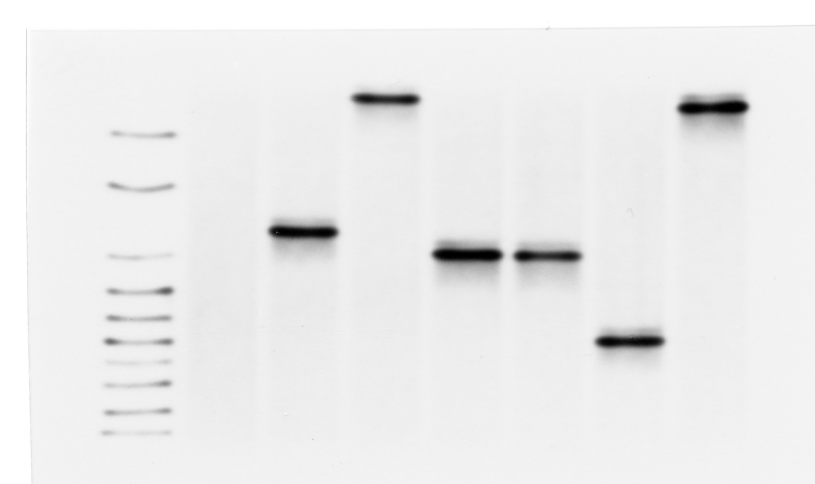

4

S13C1

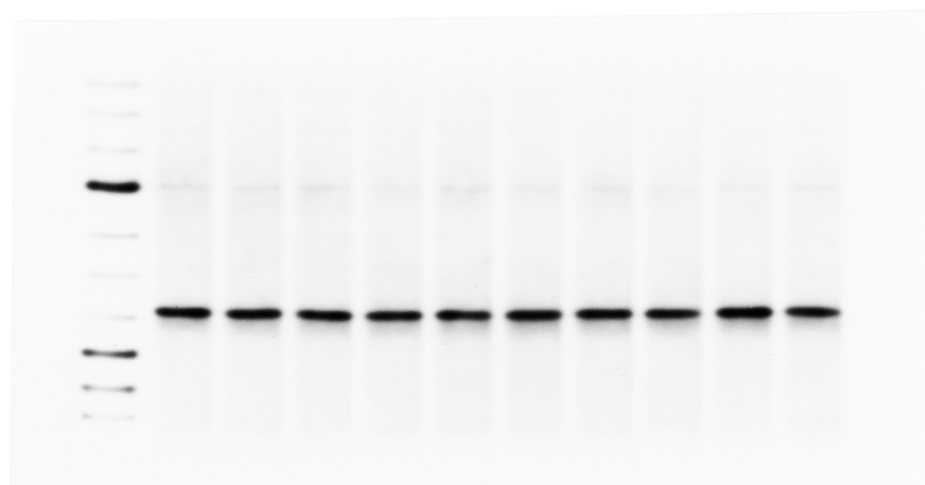

ACTB

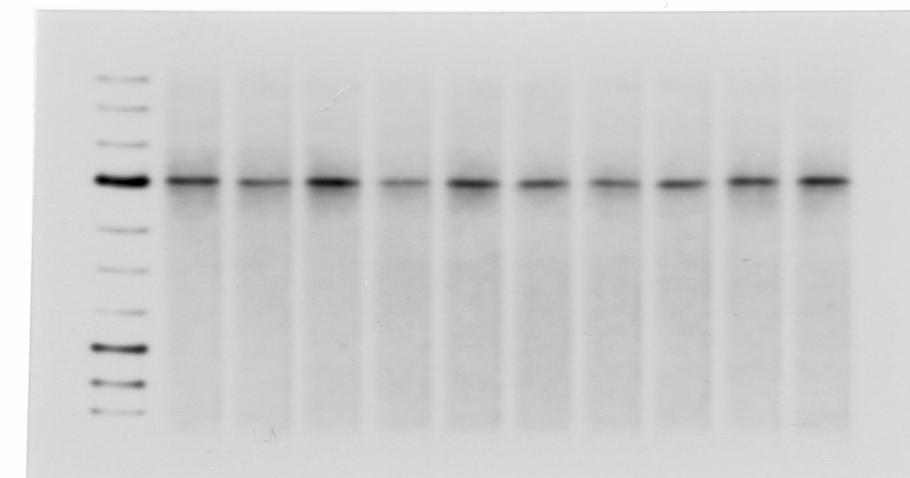

LOXL4

S13C2

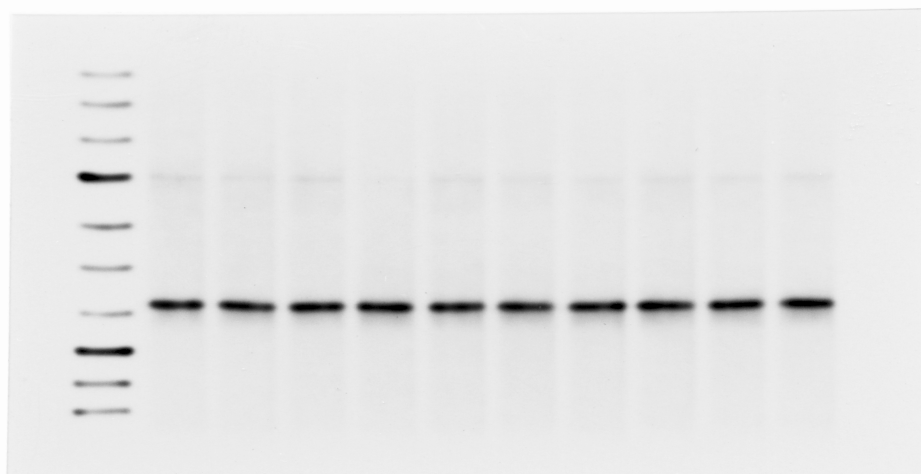

ACTB

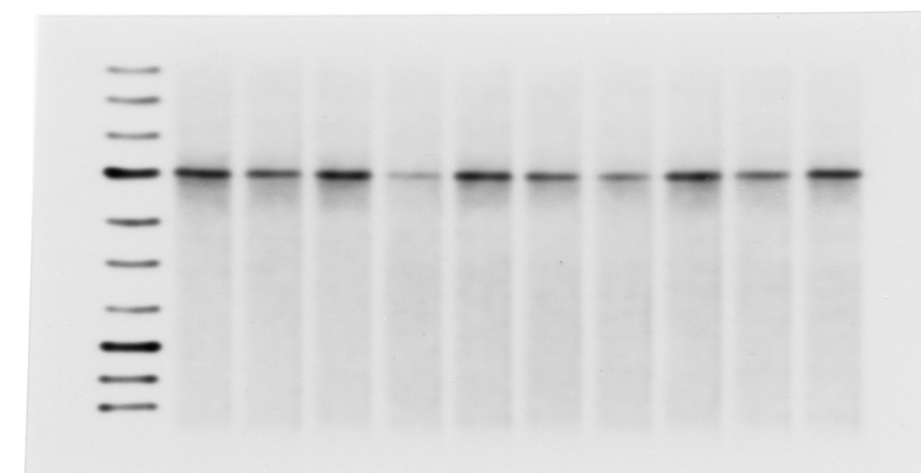

LOXL4

S4B

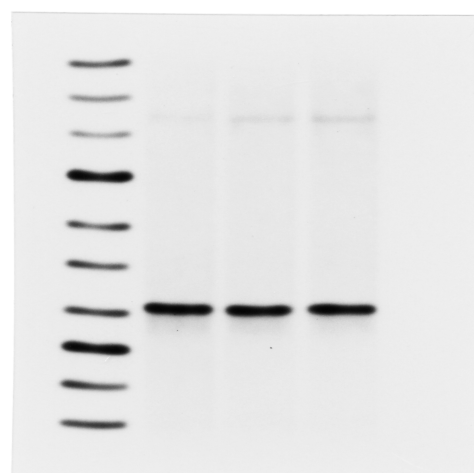

ACTB

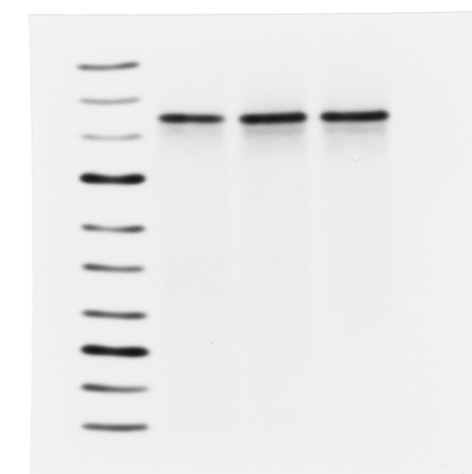

FAK1

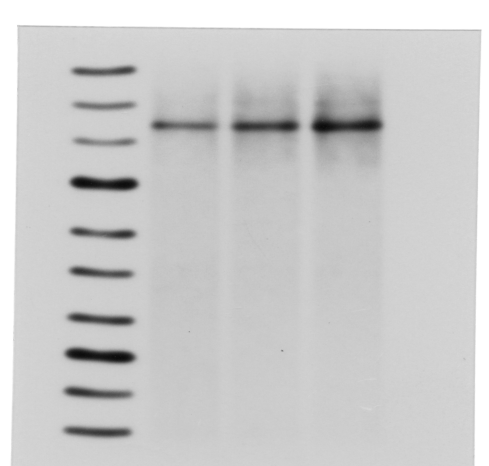

p−FAK1

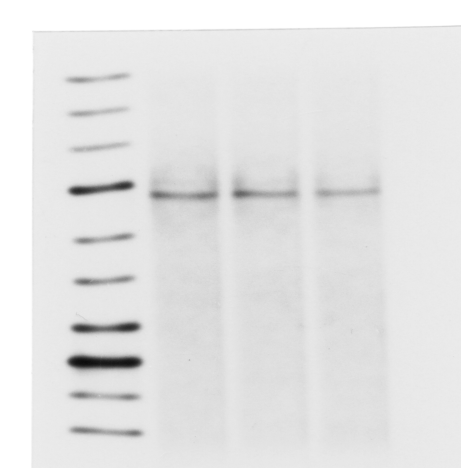

p−YAP1

S4B

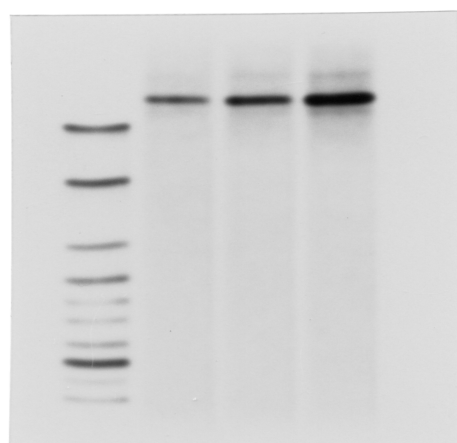

Piezo1

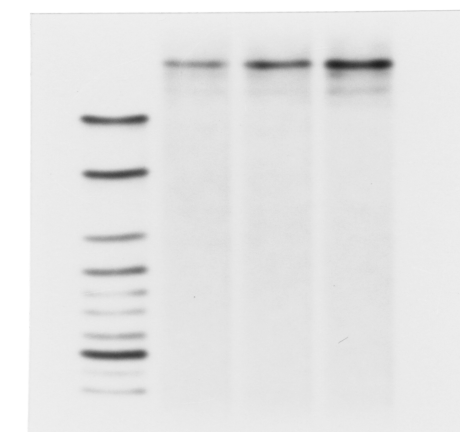

Piezo2

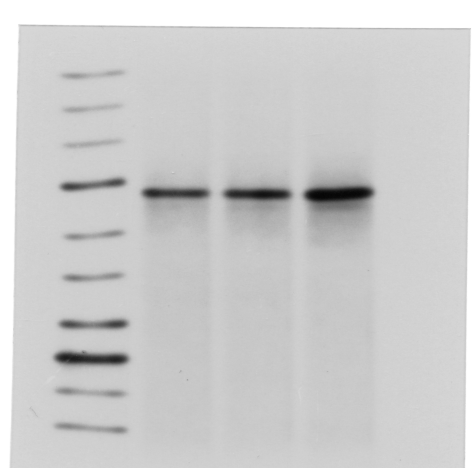

YAP1

S4G

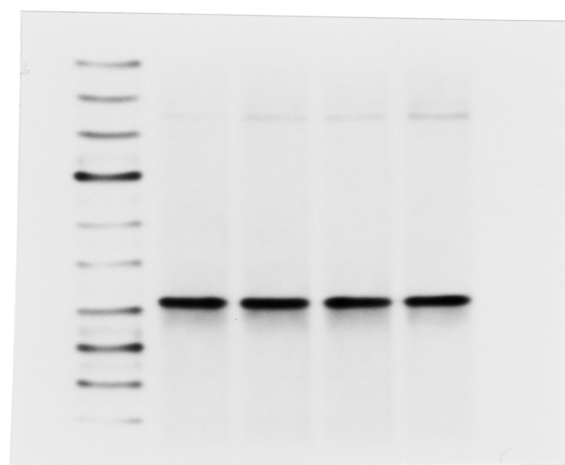

ACTB

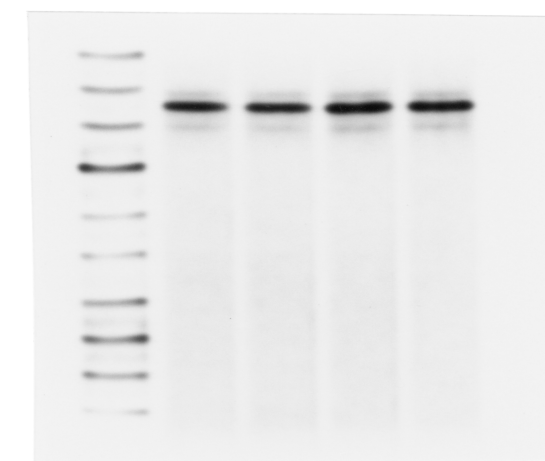

FAK1

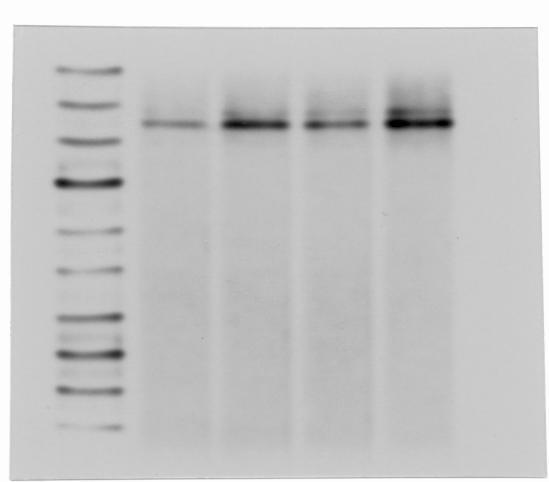

phos−FAK1

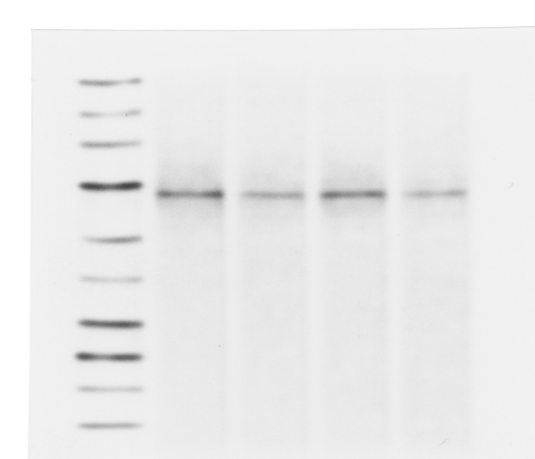

phos−YAP1

S4G

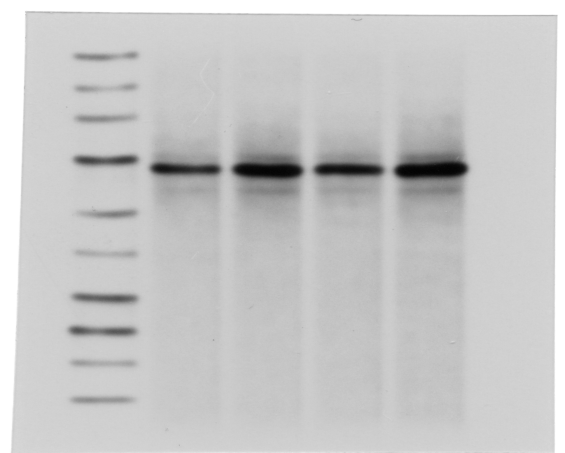

YAP1

S4I

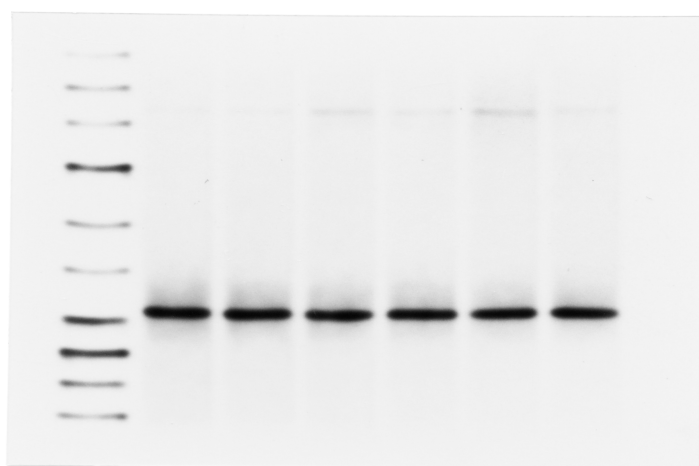

ACTB

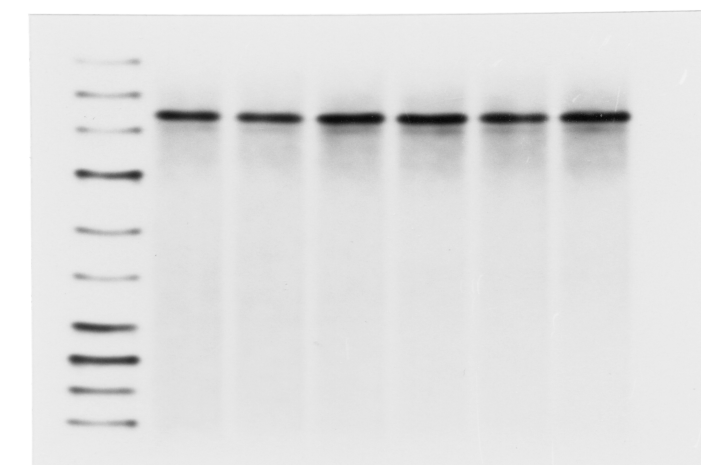

FAK1

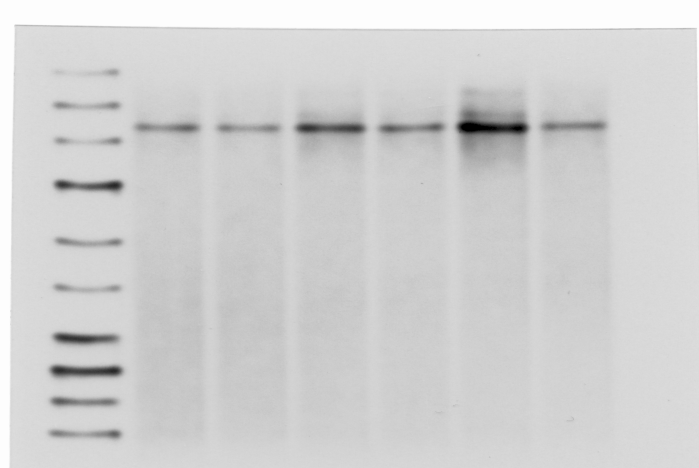

phos−FAK1

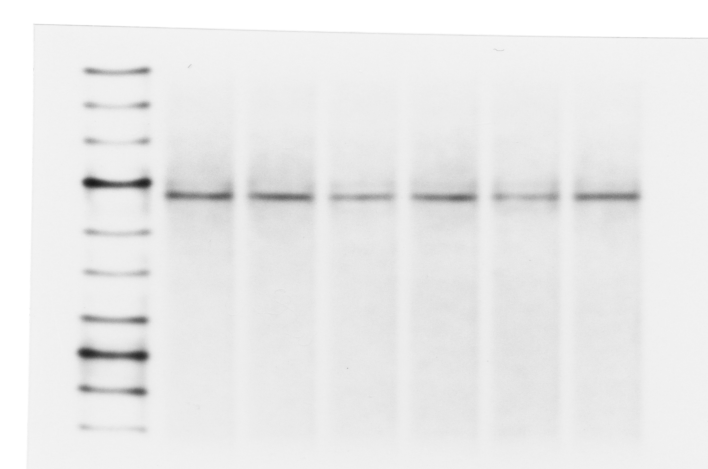

phos−YAP1

S4I

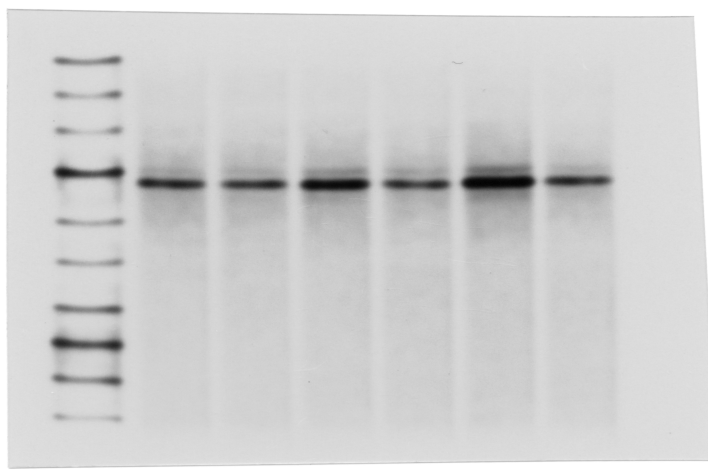

YAP1

S6C

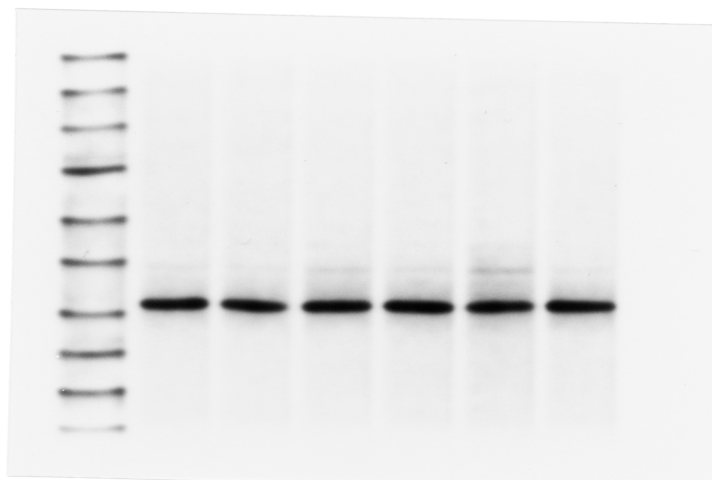

ACTB

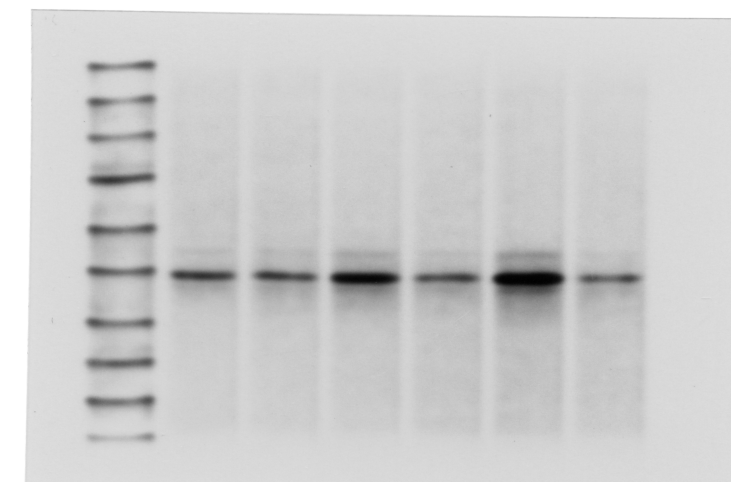

YBX1

S7A

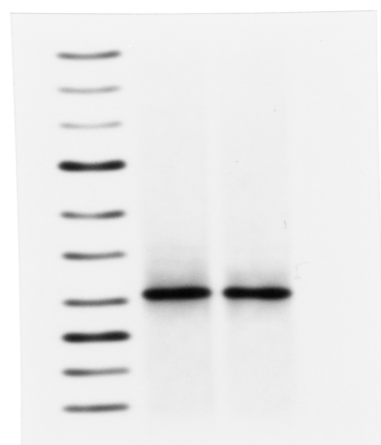

ACTB

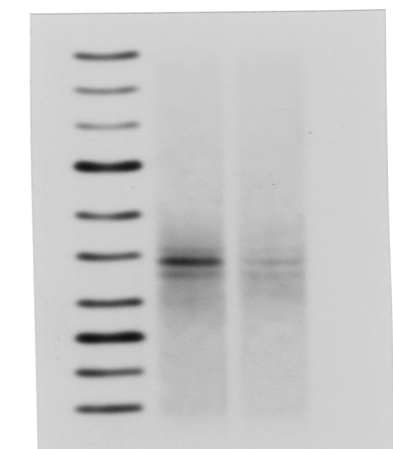

YBX1

S9D

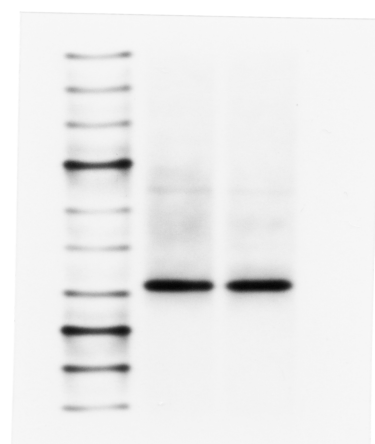

ACTB

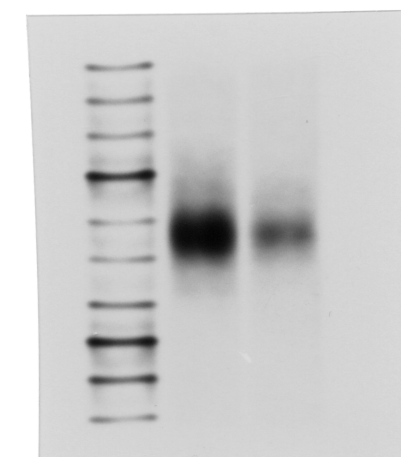

PD1

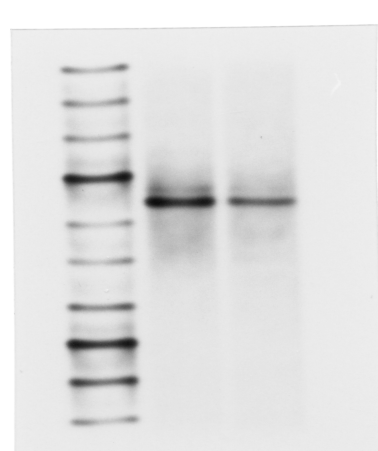

TIM3

Supplementary File 3. Raw data
